# Identification of Microorganisms that Bind Specifically to Target Materials of Interest Using a Magnetophoretic Microfluidic Platform

**DOI:** 10.1021/acsami.2c15192

**Published:** 2023-02-27

**Authors:** Song-I Han, Deborah A. Sarkes, Margaret M. Hurley, Rebecca Renberg, Can Huang, Yuwen Li, Justin P. Jahnke, James J. Sumner, Dimitra N. Stratis-Cullum, Arum Han

**Affiliations:** † Department of Electrical and Computer Engineering, Texas A&M University, College Station, Texas 77843, USA; ‡ Biotechnology Branch, U.S. Army Combat Capabilities Development Command (DEVCOM), Army Research Laboratory (ARL), Adelphi, Maryland 20783, USA; § Department of Biomedical Engineering, Texas A&M University, College Station, Texas 77843, USA; ∥ Department of Chemical Engineering, Texas A&M University, College Station, Texas 77843, USA

**Keywords:** material-binding microorganism and peptide, microfluidic screening platform, magnetic separation, bacterial surface display library, environmental microorganism screening

## Abstract

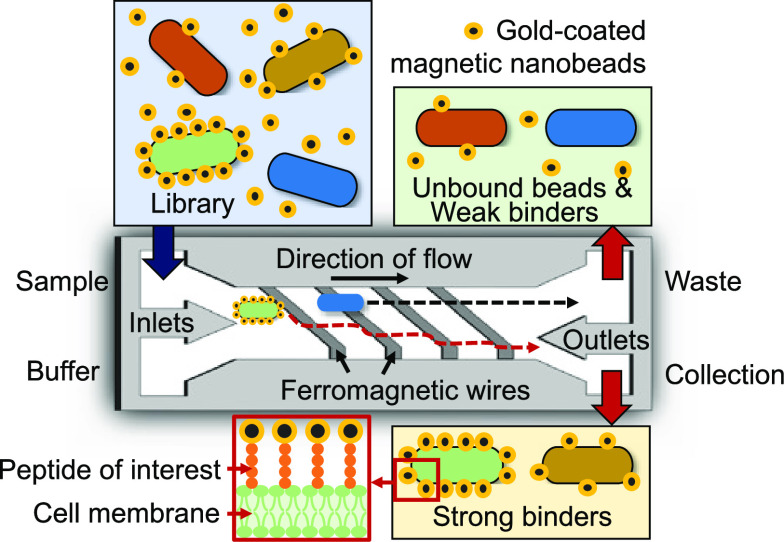

Discovery of microorganisms and their relevant surface peptides that specifically bind to target materials of interest can be achieved through iterative biopanning-based screening of cellular libraries having high diversity. Recently, microfluidics-based biopanning methods have been developed and exploited to overcome the limitations of conventional methods where controlling the shear stress applied to remove cells that do not bind or only weakly bind to target surfaces is difficult and the overall experimental procedure is labor-intensive. Despite the advantages of such microfluidic methods and successful demonstration of their utility, these methods still require several rounds of iterative biopanning. In this work, a magnetophoretic microfluidic biopanning platform was developed to isolate microorganisms that bind to target materials of interest, which is gold in this case. To achieve this, gold-coated magnetic nanobeads, which only attached to microorganisms that exhibit high affinity to gold, were used. The platform was first utilized to screen a bacterial peptide display library, where only the cells with surface peptides that specifically bind to gold could be isolated by the high-gradient magnetic field generated within the microchannel, resulting in enrichment and isolation of many isolates with high affinity and high specificity toward gold even after only a single round of separation. The amino acid profile of the resulting isolates was analyzed to provide a better understanding of the distinctive attributes of peptides that contribute to their specific material-binding capabilities. Next, the microfluidic system was utilized to screen soil microbes, a rich source of extremely diverse microorganisms, successfully isolating many naturally occurring microorganisms that show strong and specific binding to gold. The results show that the developed microfluidic platform is a powerful screening tool for identifying microorganisms that specifically bind to a target material surface of interest, which can greatly accelerate the development of new peptide-driven biological materials and hybrid organic–inorganic materials.

## 1 Introduction

Identification of microorganisms that specifically bind to a target material of interest is of significant interest for a wide range of applications, including the development of new biological materials and new hybrid organic–inorganic materials.^1−4^ Identification of unique surface peptides that contribute to this specific material binding is also of substantial interest.^5−7^ Biopanning, a method that allows affinity-based selection of microorganisms that bind to a target material of interest utilizes the simple principle that microorganisms that do not bind to the target material or bind only weakly can be easily washed off, while those that have strong affinity to the target materials remain bound on the surface after washing. This is most commonly achieved through the following three steps: (1) co-incubation of a microbial library with a target material of interest, (2) stringent washing of the material surface to remove unbound or weakly bound cells, and (3) collection and re-growth of the remaining strongly bound cells. These three stepsare then repeated multiple rounds (typically three to five rounds) to enrich the cell population and therefore obtaining cells that strongly bind to the target surface of interest with high affinity. In this procedure, the most important step that can influence the result is the wash step, where tightly controlled washing conditions that determine the degree of shear stress applied to the cells are critical in successfully identifying microorganisms that show both strong affinity and high specificity toward the target material of interest.

Conventional washing methods typically involve placing the microbial-attached substrate (i.e., target material) into a container and swirling the container on an orbital shaker to generate shear stress. Using this method, several studies have demonstrated the discovery of target-binding microorganisms and their associated peptides that most likely influence the binding affinity.^8−10^ However, precisely controlling the shear stress level is not possible in this method, since different levels of shear stress are generated in an orbital shaker depending on the location of the cell-attached substrates in the container and how they are placed.^11^ Thus, even though controlling the overall applied shear stress levels can be achieved by adjusting the rotation speed of the orbital shaker, applying a well-controlled and stringent washing condition is difficult.

To overcome these limitations, several microfluidic systems have been developed where the shear stress level can be precisely and accurately controlled by controlling the fluid flow in a microfluidic channel, achieving stringent and well-controlled wash conditions.^12−23^ Here, different shear stress levels in a microchannel can be readily generated by adjusting the flow rate using a syringe pump. However, a critical limitation of such a microfluidic biopanning system is that high shear stress cannot be easily produced due to high pressure buildup when applying a high flow rate in a relatively small microfluidic channel, limiting its utility in screening and identifying strong binders. In addition, measuring the binding strength of microorganisms to the target surface using such microfluidic systems is also quite time-consuming. To overcome these limitations, recently a centrifugal force-based microfluidic biopanning platform was developed.^24^ This microfluidic device consists of a simple straight microfluidic channel bound to a glass substrate on which a target material of interest is deposited, and by rotating the device on a spin coater, a centrifugal force-driven flow is generated within the microchannel. Using this simple setup, a very broad range of shear stress level (from 13 to 491 dyne cm^–2^, significantly higher than what can typically be generated using a syringe pump setup (3 dyne cm^–2^)^13^) can be readily generated by simply changing the rotation speed of the spin coater. This system was successfully utilized to conduct four rounds of biopanning with a bacterial peptide display library in order to identify microorganisms that specifically bind to gold or indium tin oxide (ITO). Although quite successful, significant improvements can be made to the microfluidics-based biopanning method to minimize the number of microfluidic biopanning rounds needed, which can further reduce the time and effort needed in identifying microorganisms that can specifically bind to target materials of interest. Additionally, some applications may require peptides with a more narrow range of affinity, so there is an interest in sorting microorganisms by their affinity during the screening process.

In this report, we combined the use of magnetic nanobeads coated with a target material of interest, namely, gold, and a magnetophoretic microfluidic cell separation platform to continuously sort cells that specifically bind to the target material of interest. Here, microorganisms that show highly specific affinity and strong binding to the target material will be bound to the magnetic nanobeads. The separation of microorganisms by using conventional magnetic separation techniques is quite challenging due to their small size and weak magnetic force. The developed device has ferromagnetic wires formed and inlaid underneath the microchannel where a high-gradient magnetic field can be generated, thus the magnetic nanobead-coated microorganisms can then be sorted with high separation efficiency and high sensitivity. A continuous separation method is applied, thus wash steps are not required, since only the cells with affinity against the target material will be harvested, while unbound magnetic nanobeads and cells with weak affinity to the target materials can be removed through the discard outlet. The system was first demonstrated by screening the previously characterized eCPX 3.0 library that displays unconstrained 15-mer peptides on the bacterial cell surface^25,26^ and isolating cells expressing peptides exhibiting high affinity to gold, a target material with many applications of interest, including bioelectronics, using only a single round of isolation. Second, to assess whether this method can be used to screen the extremely diverse environmental microorganisms existing in soil, we harvested environmental microorganisms from soil samples and screened them with the developed microfluidic system, which resulted in several microorganisms that show strong and specific binding to gold. These results show that the developed magnetophoretic microfluidic platform enables high-throughput, high-sensitivity, and high-efficiency discovery of microorganisms that show strong and specific affinity to a target material of interest. Since magnetic nanobeads can be coated with various target materials of interest, this platform and method has broad utility. Importantly, the success in isolating microorganisms from environmental samples shows the feasibility of tapping into the extremely vast diversity of environmental microorganisms, which can provide a new source of microorganisms that can be utilized to create new hybrid materials, or for identifying different microorganism-material binding mechanisms.

## 2 Results and Discussion

### 2.1 Design and Working Principle of the Magnetophoretic Microfluidic Platform in Separating Microorganisms that Have Strong Affinity to Target Materials of Interest

**Figure 1:**
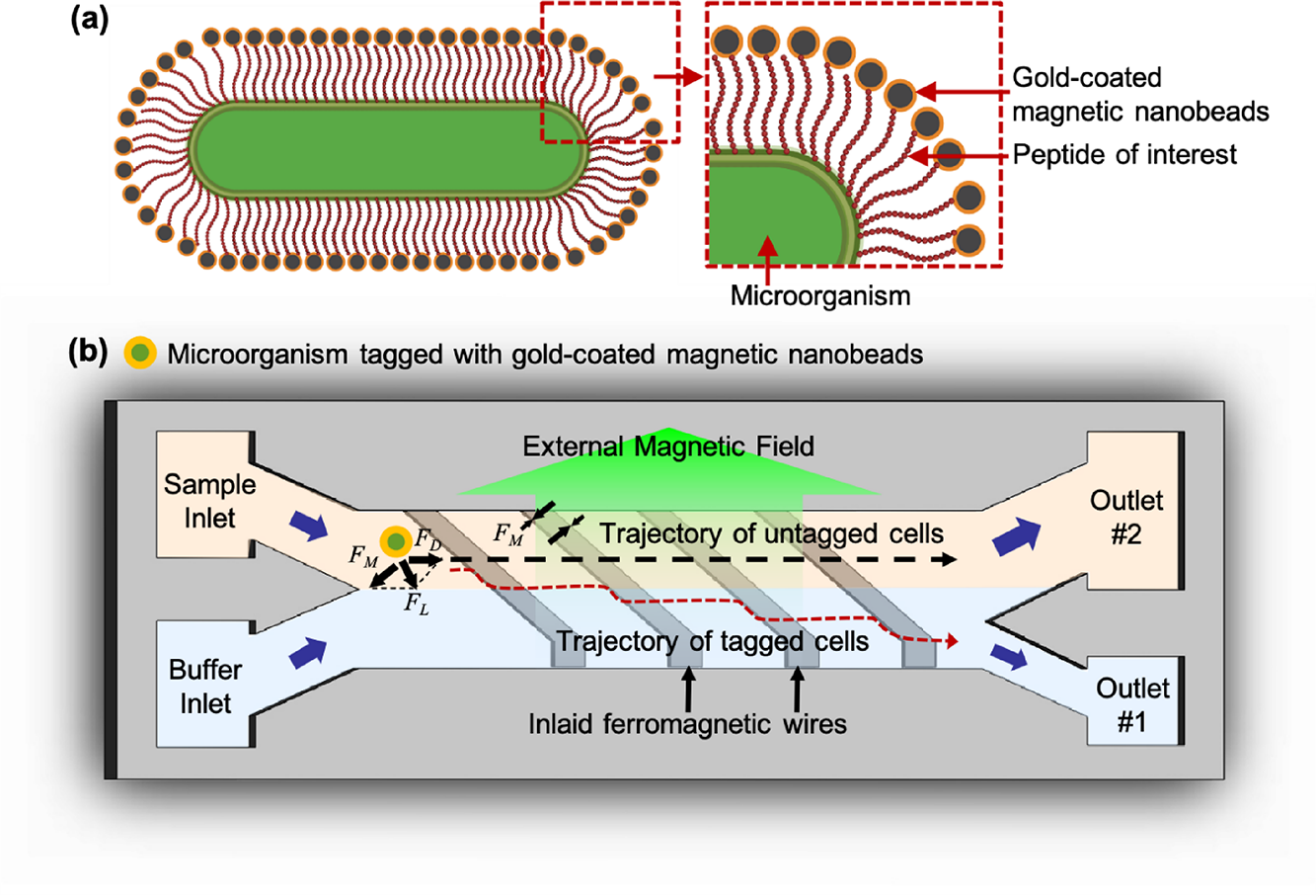
(a) Microorganism tagged with gold-coated magnetic nanobeads using the peptide of interest displayed on the cell surface. (b) Schematic showing the design of the magnetophoretic microfluidic platform for biopanning-based library screening. A stack of magnet was placed underneath the device, resulting in the high-gradient magnetic field to be generated around the integrated ferromagnetic wire. Microorganisms with weak binding to gold, not tagged with gold-coated magnetic nanobeads, flown along the main flow stream and then discarded into outlet #2. Microorganisms with strong binding to gold, tagged with gold-coated magnetic nanobeads, move laterally along the edge of the wire and separated into outlet #1. For iterative biopanning-based library sorting, the collected cells from outlet #1 were used as the starting culture for the next round of sorting.

Figure 1 shows the design and working principle of the device, and Figure S1 shows the pictures of the device and the microchannel. Cells and magnetic nanobeads coated with target materials of interest (in this case gold) are first co-incubated. Here, microorganisms that exhibit strong affinity to gold will lead to the magnetic nanobeads binding to the surface of the microorganisms (Figure 1a), while those showing little or no affinity will have few, if any, magnetic nanobeads bound to the microbial cell surface. These magnetic nanobead-tagged cells, behaving as paramagnetic particles, can then be separated using a magnetophoretic microfluidic device. The diameter of the magnetic nanobeads is 20 nm to avoid the sedimentation after the cells are tagged with the gold-coated magnetic nanobeads. The developed magnetophoretic microfluidic platform has two inlets, two outlets, and a downward-tilted ferromagnetic wire array patterned on the bottom glass substrate (Figure 1b). A magnet placed underneath the device results in a high-gradient magnetic field to be generated along the tilted-angle (5.7°) ferromagnetic wires, as shown in the COMSOL simulation result (Figure S2). This is because when an external magnetic field is applied to the wire, the external magnetic field is deformed near the wire, resulting in a high-gradient magnetic field to be generated at the edge of the wires. The separation of microbial cells is quite challenging due to their small size, but the generated high-gradient magnetic field can generate strong magnetic force to cells regardless of the size; thus, the developed platform can provide high specificity and high separation efficiency. The flow rate for sample and buffer injection was set to be the same flow rate, and continuous laminar flow was generated along the microchannel. When the cells tagged with gold-coated magnetic nanobeads pass over the wire, they will experience both a magnetic force (*F*_M_) and a hydrodynamic drag force (*F*_D_). The lateral magnetic force (*F*_L_) applied to the cells will be a vector sum of the magnetic force and the drag force.^27^ Thus, under an externally applied magnetic field, cells tagged with gold-coated magnetic nanobeads will move along the tilted wire and then be separated into outlet #1. On the other hand, cells that have weak affinity to gold will not bind to gold-coated magnetic nanobeads and thus will flow straight along the sample flow streamline unaffected by the magnetic field and then flow out through outlet #2.

**Figure 2:**
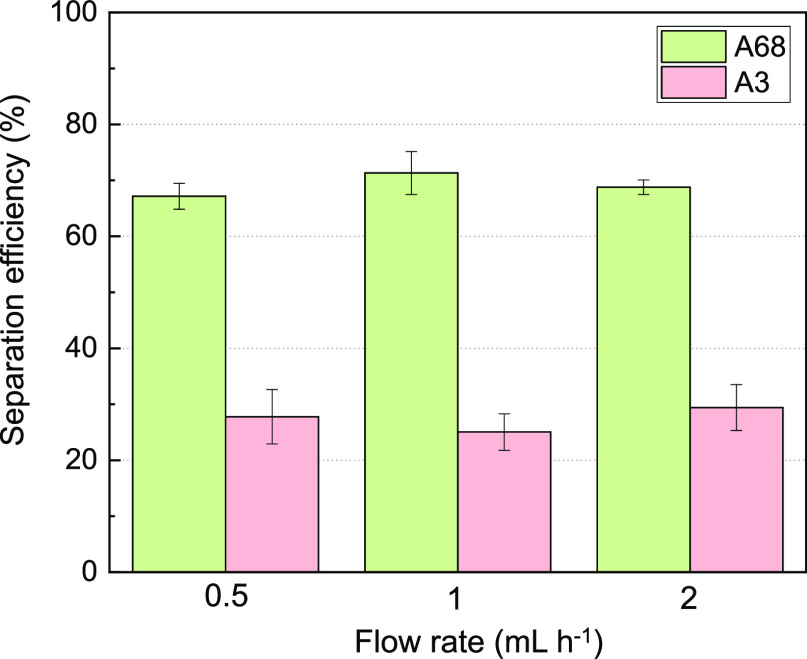
Separation efficiency of A68 and A3 variants at different flow rates of 0.5, 1, and 2 mL h^–1^. The average of separation efficiency was defined as , and all measurements were taken in triplicate.

The capability of the developed magnetophoretic microfluidic device in separating cells having different affinities for gold was assessed using two different *Escherichia coli* strains (A68 and A3) known to have different affinities toward gold, isolated from a previous work,^24^ along with several different negative controls (Table S1). In two separate experiments, cells were injected into the device and then cells flowing out from outlet #1 were collected. The number of cells collected from this outlet #1 was quantified by plating them on an agar plate and counting the number of colonies formed (Table S2). In the case of the A68 strain (known to have high affinity to gold), 2.3 × 10^8^ ± 5 × 10^6^ colonies (about 69% of the population) were obtained from outlet #1 while approximately 3.3 × 10^8^ cells were flown through the device (flow rate: 2 mL h^–1^, separation duration: 10 min). In the case of the A3 strain (known to have very weak affinity to gold), 9.7 × 10^7^ ± 1.4 × 10^7^ colonies (about 29% in the population) were obtained from outlet #1 while approximately 3.3 × 10^8^ cells were flown through the device (flow rate: 2 mL h^–1^, separation duration: 10 min). Based on this, the separation efficiency (defined as ) of the A68 strain was determined to be 2.4-fold higher compared to that of the A3 strain. This clearly shows that cells with high affinity to gold can be isolated using this method. To determine the maximum flow rates that will still show a reliable separation trend between the two strains having different affinity to gold, the experiments were conducted at three different flow rates of 0.5, 1, and 2 mL h^–1^ (the same flow rate for both sample and buffer injection) (Figure 2). We confirmed that the separation efficiency (69% of A68 vs 29% of A3) and the separation trend (2.4-fold difference) between the two strains was similar regardless of the flow rate used. Based on this characterization, in the subsequent library sorting experiment, a flow rate of 2 mL h^–1^ was used for the microfluidic separation system. Even though A68 is known to have high affinity to gold, the separation efficiency is about 70%. This might be caused by a moderate degree of cell aggregation, which can then be more easily stuck on the ferromagnetic wire in the microchannel during the separation process, or stuck in the syringe before they can be injected into the microchannel.

### 2.2 Screening the eCPX 3.0 Peptide Display Library to Isolate Specific Peptide Binders against Gold

The developed microfluidic device was used for conducting four sequential rounds of sorting using the eCPX 3.0 bacterial display library that contains ∼10^10^ individual library members to enrich isolates displaying peptides with high affinity to gold. Approximately 5 × 10^11^ cells suspended in 3 mL of phosphate-buffered saline (PBS) were injected into the microdevice through the sample inlet. Cells were collected from outlet #1 (expected to have gold magnetic nanobeads attached to the cells due to their strong affinity to gold), cultured for expansion, and then used as the starting population for the next round of sorting.

The indirect binding assay described in our previous study^24^ was used to quantify the relative affinity of the sorted population between each sorting round; the collected samples were quantified by colony counting on LB agar plates with antibiotics. The population-level affinity increased from approximately 21-fold (NC-P2X (Table S1) vs the sorted population, round 1) to 24-fold (round 2), and then to 29-fold (round 3), and finally to 32-fold (round 4). We observed that the affinity of cells to the gold increased from round to round, as compared to the negative control NC-P2X cells, which clearly demonstrates that the population was enriched for high affinity binders to gold.

**Figure 3:**
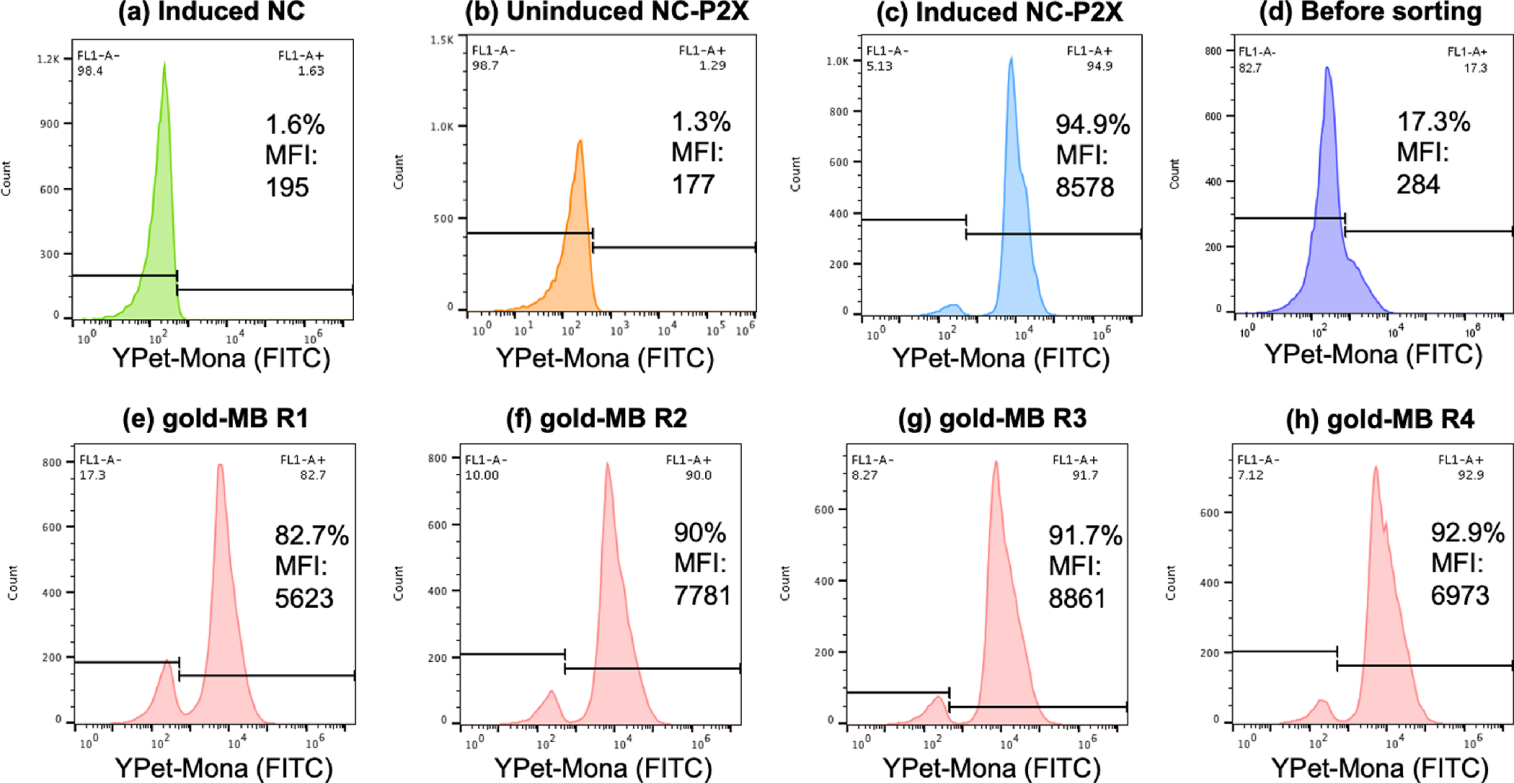
Histograms of (a) induced NC, (b) uninduced NC-P2X, and (c) induced NC-P2X with YPet-Mona staining, where the *x*- and *y*-axes indicate the intensity of YPet-Mona (FITC-A) and the number of cells counted, respectively. YPet-Mona, which corresponds to the degree of overall eCPX scaffold expression, binds to the P2X peptide displayed on the cell surface. (d) Histogram of the eCPX 3.0 library before sorting. The percentage of the P2X peptide expression of cells within the population in (e–h) sorting round 1 to round 4 was analyzed. In round 4, over 90% in the population is expressing a valid peptide, as determined by the ability to properly display the C-terminal P2X peptide.

The peptide expression level was monitored after each sorting round since the peptide expression level of the eCPX 3.0 display scaffold is also expected to increase after each round of successful enrichment as peptides with stop codons or other interruptions that prevent proper expression and folding of eCPX scaffolds are removed from the population. Monitoring the peptide expression level was conducted by assessing binding to YPet-Mona, a fusion of a yellow fluorescent protein with an SH3 domain that binds to the P2X peptide at the C-terminus of the eCPX scaffold.^28^ The fluorescence intensity of YPet-Mona therefore corresponds to the degree of overall eCPX scaffold expression and thus the peptide expression on the cell surface, which was analyzed by flow cytometry. Induced NC and uninduced NC-P2X cells were used as negative controls, and induced NC-P2X cells were used as a positive control for expression (Table S1). Here, uninduced and induced NC-P2X cells enable setting the “gates” for autofluorescence. In the case of negative control cells (NC cells induced with l-arabinose and NC-P2X cells uninduced with l-arabinose) showed very low fluorescence, meaning low or no eCPX scaffold or peptide expression, due to the lack of P2X expression on the surface of the cells, as expected (Figure 3a,b). Here, the percentage of cells falling outside of the area of autofluorescence was 1.6% of the population (median fluorescence intensity [MFI] = 195) for induced NC cells and 1.3% (MFI = 177) for uninduced NC-P2X cells. For positive control NC-P2X cells induced with l-arabinose and displaying P2X peptides at the C-terminus of the cell surface, a robust fluorescence expression was observed due to binding of YPet-Mona to the P2X peptides (Figure 3c), with 94.9% (MFI = 8578) of the population showing fluorescence.

Based on this calibration and the gate settings, the P2X peptide expression level of the eCPX 3.0 library itself was observed before iterative biopanning sorting, where only 17.5% of the population expressed the P2X peptide (Figure 3d). Next, cells recovered from each sorting round were analyzed by applying the same gate to observe their P2X peptide expression levels. As shown in Figure 3e–h, the percentage of cells displaying the P2X peptide increased through each sorting round, from 82.7% in round 1 (MFI = 5623) to 90.0% in round 2 (MFI = 7781), to 91.7% in round 3 (MFI = 8861), and finally to 92.9% in round 4 (MFI = 6973). It is to be noted that even after just the second sorting round (round 2), P2X peptide expression already exceeded 90% of the population, and even after the first sorting round (round 1), P2X peptide expression exceeded already 80% of the population. This strongly suggests that even after one to two rounds of sorting, sufficient levels of enrichment (exceeding 80%) can be achieved, demonstrating the significantly high selection/sorting efficiency of the developed microfluidic device. Compared to our previously reported centrifugal microfluidic-based biopanning approach,^24^ where it took three rounds of sorting for the P2X peptide expression of the population to even exceed 80% (from 73.2% in round 1 (MFI = 2615), to 78.4% in round 2 (MFI = 3148), to 84.6% in round 3 (MFI = 3867), and finally to 95.2% in round 4 (MFI = 4501)), this result suggests that more efficient and quicker sorting can be achieved using this novel microfluidic device.

### 2.3 Characterization of Isolated Single Colonies for Their Material-Binding Phenotypes

To analyze the sorted gold-binding isolates, cells from round 1 sorting and round 4 sorting were tested using three complementary methods:^24^ a surface-binding spot assay to confirm gold-binding affinity, flow cytometry to monitor P2X expression, and DNA sequencing to determine the displayed peptide sequence. In our previous study, we have observed that the isolated “original” colonies directly obtained from the agar plates after sorting showed a relatively low material binding level as compared to the positive control gold binder strains.^24^ Thus, a retransformation process was carried out by isolating each plasmid from the original colonies, followed by transformation of the isolated plasmids into the “Z-competent” MC1061 *E. coli* strain. This process also ensures that binding of cells to the target material is not due to mutations in the cell itself but rather due to the affinity of the peptides toward the target material. Overall, 50 colonies from round 1 and 50 colonies from round 4, total 100 colonies, were randomly picked, each isolates re-transformed, and then analyzed.

**Figure 4:**
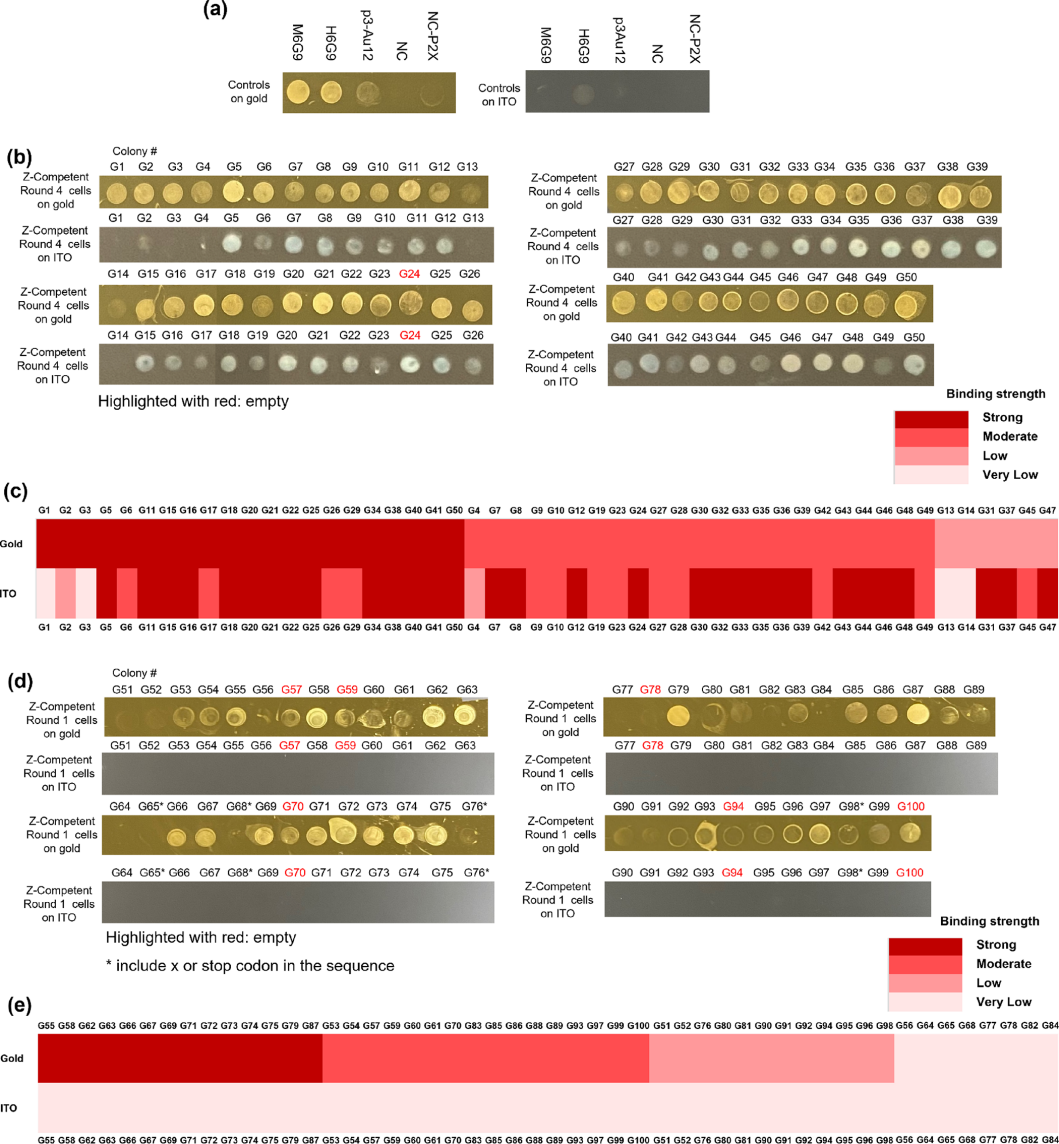
Surface binding spot assay of positive and negative controls and single colonies obtained from sorting round 1 and sorting round 4 using gold-coated magnetic nanobeads. Affinity and specificity for gold and ITO for (a) negative and positive control cells, for (b) 50 colonies obtained from sorting round 4, and for (d) 50 colonies obtained from sorting round 1 were assessed. (c, e) The degree of the affinity to gold and ITO were categorized and presented in a heat map.

First, surface-binding spot assays were conducted on gold and ITO substrates to test the affinity as well as specificity of the cells. Unless otherwise stated, M6G9, H6G9, and p3-Au12 were used as positive controls against gold (Table S1), and NC as well as NC-P2X used as negative controls (Figure 4a).^24^ The result of the surface-binding spot assay for all 50 colonies isolated from the final sorting round (round 4) is shown in Figure 4b. All colonies were classified into four categories based on the degree of the affinity to gold and/or ITO, (1) strong, (2) moderate, (3) weak, and (4) very weak, and displayed as a heat map (Figure 4c). In the case of gold binding, it is seen that 42% of the population (21 out of 50) has strong binding to gold and their binding strength is comparable to that of the positive control strains M6G9 and H6G9 (both known to have strong affinity for gold). Moderate binders occupied 46% of the population (23 out of 50), and their binding strength is slightly lower than that of M6G9 and H6G9 but higher than that of p3-Au12 (known to be a moderate binder to gold). Only 12% of the population (6 out of 50) showed weak binding to gold, but their binding strength still approached that of p3-Au12. In terms of their affinity to ITO to assess the specificity of material binding of these isolates, spot assays on ITO showed that most isolates also exhibit strong or moderate binding to ITO as well, with the binding strength comparable to that of H6G9 (known to be a strong binder to ITO), except for G1, G2, G3, G4, G13, and G14. Among these isolates, only G1 and G3 showed high affinity and high specificity to gold. The overall trend here is that after four rounds of selection, a high number of strong gold binders were isolated, but they also showed relatively strong binding to ITO as well, with only two strains showing specific binding to gold but not to ITO. This is not too surprising since our previous results showed that there were significant overlaps between the amino acid residues responsible for binding to gold and ITO,^24^ and no negative sorting for specificity was performed here. The specificity can be improved by including negative sorting steps against ITO or other materials to be excluded. This may require increasing the sorting steps beyond four rounds.

Next, to assess the results from only a single round of sorting, especially since based on the P2X expression results (Figure 3e), a single round may be sufficient to identify strong gold binders, 50 colonies were isolated from the first sorting round (round 1) and their Z-competent cells were tested through the surface-binding spot assay (Figure 4d). The degree of affinity of each colony to gold and ITO was categorized and displayed as a heat map (Figure 4e). Here, strong binders to gold occupied 28% of the population (14 out of 50), with their binding strength comparable to that of M6G9 and H6G9 (known to be strong gold binders) against gold. Moderate binders occupied 32% of the population (16 out of 50), with their binding strength comparable to that of p3-Au12 (known to be a moderate gold binder). Also, 24% of the population (12 out of 50) displayed low binding to gold, with their binding strength comparable to that of NC-P2X (known to be a weak binder to gold). Very weak binders occupied 16% of the population (8 out of 50), with their binding strength comparable to that of NC. Overall, low or very low binders accounted for 40% of the population (20 out of 50) while there were no cells with such low affinity to gold in the round 4 sorting result. When comparing the affinity to ITO, it is seen that no cells showed high binding to ITO, showing very high specificity to gold only. This is in contrast to the result seen from round 4 sorting, where most isolates showed high affinity to multi-materials (both gold and ITO). These results suggest that to obtain isolates that show only specific binding to the target material of interest (gold in this case), only a single round of sorting is actually desired, while if the purpose is to identify strong metal binders in general, regardless of their specificity, multiple rounds of sorting may be better. Alternatively, negative sorting rounds can be interspersed to exclude binders that bind to both materials.

### 2.4 Identification of Peptide Sequences from the Isolated Strains

DNA sequencing of the 100 isolates that were tested for their affinity to gold and ITO (50 from round 1 and 50 from round 4) was conducted to determine the amino acid sequence of the displayed peptides, with the result shown in Table S3, “Sequence” column. Colonies G-1 to G-50 are from round 4 sorting, while colonies G-51 to G-100 are from round 1. The levels of P2X peptide expression were also measured by assessing YPet-Mona binding to the C-terminal P2X peptide using flow cytometry (Table S3, “YPet-Mona (%)” column). The results show that most cells were confirmed to exhibit over 80% of P2X peptide expression. Peptide expression was poor only in four of the colonies tested (G65, G68, G76, G84, all from round 1 selection), which contained empty vectors that expressed eCPX but no N-terminal peptide and no stop codons. Even in the absence of truncations, unknown residues, or stop codons, the G84 isolate showed a much lower P2X expression (2.17%), indicating that it may be toxic to the cell, although having a higher arginine content was also reported in our previous work to inhibit eCPX expression in general.^24,29,30^ From the round 4 sorting, out of 50 isolates only one isolate (G24) showed an empty plasmid with 95.7% P2X peptide expression. For the round 1 sort, out of 50 isolates 6 isolates (G57, G59, G70, G78, G94, and G100) showed empty plasmids with over 80% P2X peptide expression. Despite the absence of a unique peptide at the N-terminus, G24 showed moderate affinity to gold and strong affinity to ITO, and G57, G59, and G70, while G100 showed moderate affinity to gold, which may have been affected by the binding of the variant itself to gold or ITO. The affinity of G78 and G94 to gold is weak and approaches the binding strength of NC-P2X to gold, which may be due to the binding of the P2X peptide itself to gold rather than due to the N-terminal peptide’s affinity for gold and ITO. Isolates G10, G32, G44, and G49 showed truncation to less than 15 amino acid residues, but they still produced valid peptides and showed strong affinity to gold and ITO in the surface-binding spot assay. Isolates G86 and G92 were also truncated to less than 15 amino acid residues, but G86 showed moderate binding to gold and G92 showed weak binding to gold. Isolate G64 expressed only seven amino acid residues, and its binding strength to gold is very weak.

**Figure 5:**
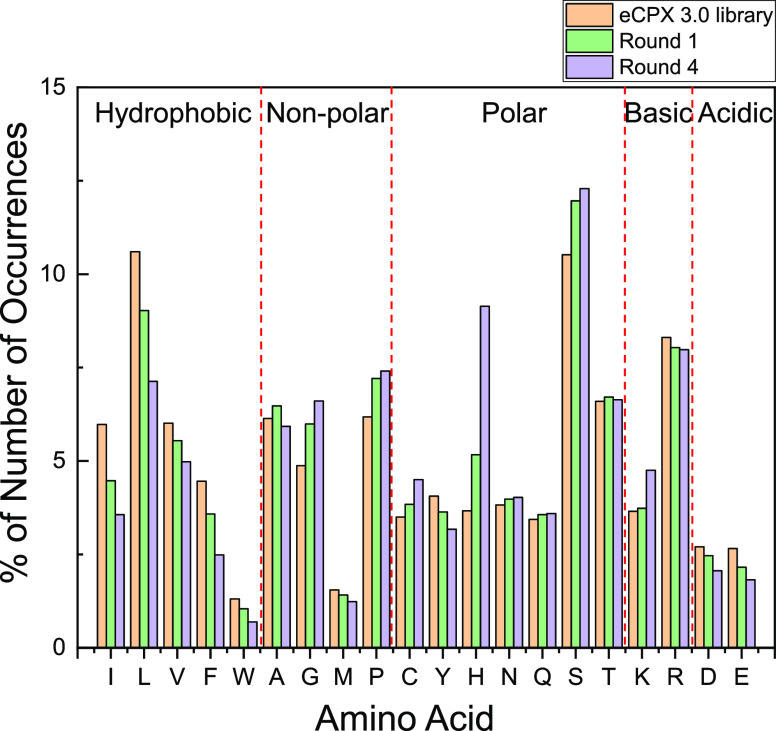
Analysis of amino acid frequency in the isolated 15-mer peptides from biopanning the eCPX 3.0 library for peptides with gold affinity using the magnetophoretic microfluidic platform. The frequency of each amino acid within the eCPX 3.0 library (orange bars) is compared to the frequency within the entire round 1 population after sorting using gold-coated magnetic nanobeads (green bars) and the entire round 4 population after sorting (purple bars). Total occurrence of all 20 amino acids was normalized by the total number of residues.

Next-generation sequencing (NGS) was performed to investigate trends in the amino acid content of the peptide sequences related to gold binding. Based on NGS data, the frequency of amino acid occurrence in the eCPX 3.0 library and the samples from the gold round 1 and round 4 sorting was examined (Figure 5) using a previously described analysis method.^24^ In brief, the percentage of the number of amino acid occurrences was categorized into hydrophobic, non-polar, polar, basic, and acidic amino acids. Here, only 15-mer peptide and valid sequences without blank inserts, frame shifts, and stop codons were used (Table S4). Figure S3 shows the distribution of length of valid display peptide inserts in round 4 sorting, demonstrating that most peptides displayed were 15-mers. The raw count of amino acid occurrence along the length of valid 15-mers in the eCPX 3.0 library^24^ and round 4 sorting are shown in Figure S4. In round 4 sorting (Figure S4b), histidine (H) shows a distinct preference for the N-terminal end over the C-terminal end of the insert sequence. This is in contrast to the profile for H in the eCPX 3.0 library, which has no notable trend depending on the insert length. Several amino acids, particularly leucine (L), lysine (K), and arginine (R), showed a preference for the insert ends by round 4 sorting relative to the peaks in the middle of the insert shown in the native eCPX3.0 profiles for these same residues. Proline (P), in contrast, changes from no real preference along the insert length in the eCPX3.0 library to demonstrating a marked preference for the middle of the insert by round 4 sorting, possibly to accommodate the concomitant kink in the insert structure.

Analysis of the trends in amino acid occurrence between the native eCPX3.0 library compared to multiple rounds of the magnetophoretic microfluidic-based biopanning method (Figure 5) yields interesting information on the roles of various residues in the binding process. Overall, the percentages of hydrophobic amino acids and acidic amino acids decrease and the percentage of polar amino acids, especially histidine (H), increases through the multiple sorting rounds. We particularly noted increases in glycine (G), proline (P), histidine (H), and serine (S), which have been previously shown to play a role in gold binding.^28,31,32^ The percentage of glycine (from 4.9% in the unsorted eCPX 3.0 library to 6% in round 1 and then to 6.6% in round 4), proline (from 6.2 to 7.2 and then to 7.4%), and serine (from 10.5 to 12 and then to 12.3%) increased through the multiple rounds of enrichment. Specifically, the percentage of histidine increased through the multiple sorting rounds, where the percentage of histidine in round 1 was 1.5% higher, and histidine in round 4 sorting was 5.5% higher, compared to the percentage of histidine in the unsorted eCPX 3.0 library (Figure 5). Although universal design rules for gold binding have remained elusive, these results are consistent with the literature.^33−35^ Histidine (H) has been flagged as a potential gold binder in our previous work as well as in early attempts to develop design rules for inorganic binding peptides.^24,33^ The increase in glycine (G) is also consistent with these design rules, as well as in simulation studies flagging the importance of peptide flexibility in the binding process.^34^ Serine (S) has been postulated to play a role in gold binding both experimentally^35^ and from simulation, where it has been proposed to facilitate motion to the surface and function as an anchor point.^34^ The situation is similar for proline,^34,35^ where it is proposed to enhance sampling and exploration of the binding surface. The percentage of cysteine (C), known to have high affinity to gold, is not high and only slightly increases over the sorting rounds (from 3.5 to 3.8 and then to 4.5%). We note that this is in contrast to our previous results using a centrifugal microfluidic platform for sorting,^24^ where large increases were seen in cysteine (C). The source of this difference is unknown at this point and may be attributable to differences in the two protocols or binding modes. It is also of interest to note that even though methionine (M) has been thought to have high affinity to gold,^33,34^ it showed a mild decrease over the sorting rounds (from 1.5 to 1.4 and then to 1.2%). Similarly, the basic residue lysine (K) was heavily expressed in round 4 sorting, while the putative binder arginine (R)^35^ was suppressed over the sorting rounds. Generally, the percentage of hydrophobic and acidic residues is lower when compared to the unsorted eCPX 3.0 library, indicating that these residues are gradually suppressed through the multiple rounds of enrichment for gold binding peptides.

Valid sequences of isolated from the round 4 sorting against gold were subjected to additional analyses. Searches for motifs were performed both on the whole sequences (via WebLogo)^36,37^ and on 4-mer sequence fragments via Linux and python tools. A heat map of co-occurrence of pairs of amino acids in the round 4 gold sort was produced and is provided in Figure S5. No overarching motif was found for the whole sequences, and analysis of 4-mer fragments within the library demonstrated heterogeneous occurrence of highly expressed amino acids consistent with the amino acid profiles just discussed. The heat map demonstrates a homogeneous preponderance of serine (S) co-expression with other binding residues, consistent with its putative role as a binding anchor.

Valid 15-mer peptide sequences from round 1 and round 4 sorting against gold were analyzed and arranged in order of their frequency of occurrence within the library. Table S5 shows the list of top 100 sequences and their frequency. Note that no amino acid sequences within the 100 isolates obtained from the round 1 and round 4 sorting matched the top 100 sequences from the NGS analysis of the round 1 and round 4 sorting, which is consistent with the previous study,^24^ meaning that the diversity of amino acid residues in 15-mer peptides sorted from the library sorting using gold is extremely high, which may be affected by the binding of the P2X peptide itself to gold. Additionally, we note that the top 10 most frequent sequences for this sort do not occur among the most frequently occurring sequences from our centrifugal microfluidic flatform sort, and vice versa. This is consistent with the differences noted in the amino acid profiles and again suggests a possible difference in experimental protocols and binding mode between the two methods.

We also note the consistency of histidine occurrence for ITO binders in the current work. In our previous study,^24^ we discovered that histidine in isolates obtained from round 4 sorting against ITO was dominant among the amino acid residues, where the percentage of histidine in isolates from round 4 sorting was 5.7% higher compared to that in the unsorted eCPX 3.0 library. In this study, 31 variants among 50 strains (∼65%) from round 4 sorting showed strong binding to both gold and ITO, which may be mostly affected by the heavy expression of histidine. However, histidine expression in the variants obtained in round 1 is slightly higher than that of the unsorted eCPX 3.0 library, but these variants showed very low affinity to ITO based on the surface-binding spot assay result (Figure 4d).

### 2.5 Discovery of Soil Microorganisms with Strong Affinity for Gold

So far, biopanning of microorganisms to identify strong binders to target metals of interest has only been conducted using synthetic microbial libraries. Soil provides a rich source of extremely diverse microorganisms, thus we sought to isolate and identify microorganisms from soil that show strong affinity to the target material of interest. To achieve this, the developed microfluidic device was used to isolate soil microorganisms with strong binding affinity and specificity to gold.

Two soil samples, loam soil and feedlot soil, were collected from different regions in the United States (loam soil taken from outside of the new warming experimental plots created in 2009 from 34°58′45″ N, 97°31′15″ W, and feedlot soil taken from 30°31′12.75″ N, 96°34′52.97″ W, and microorganisms were extracted from these soil samples using a soil microorganism extraction protocol recently developed through a collaboration.^38−40^ Approximately 5 × 10^7^ soil microorganisms suspended in 3 mL of PBS were co-incubated with 100 μL of gold-coated magnetic nanobeads (10^13^ magnetic nanobeads) for 1 h, followed by flowing them through the microfluidic device presented here. All conditions used here were identical to those described above. Cells flowing out of outlet #1 were collected and plated on an agar plate for off-chip analyses.

**Figure 6:**
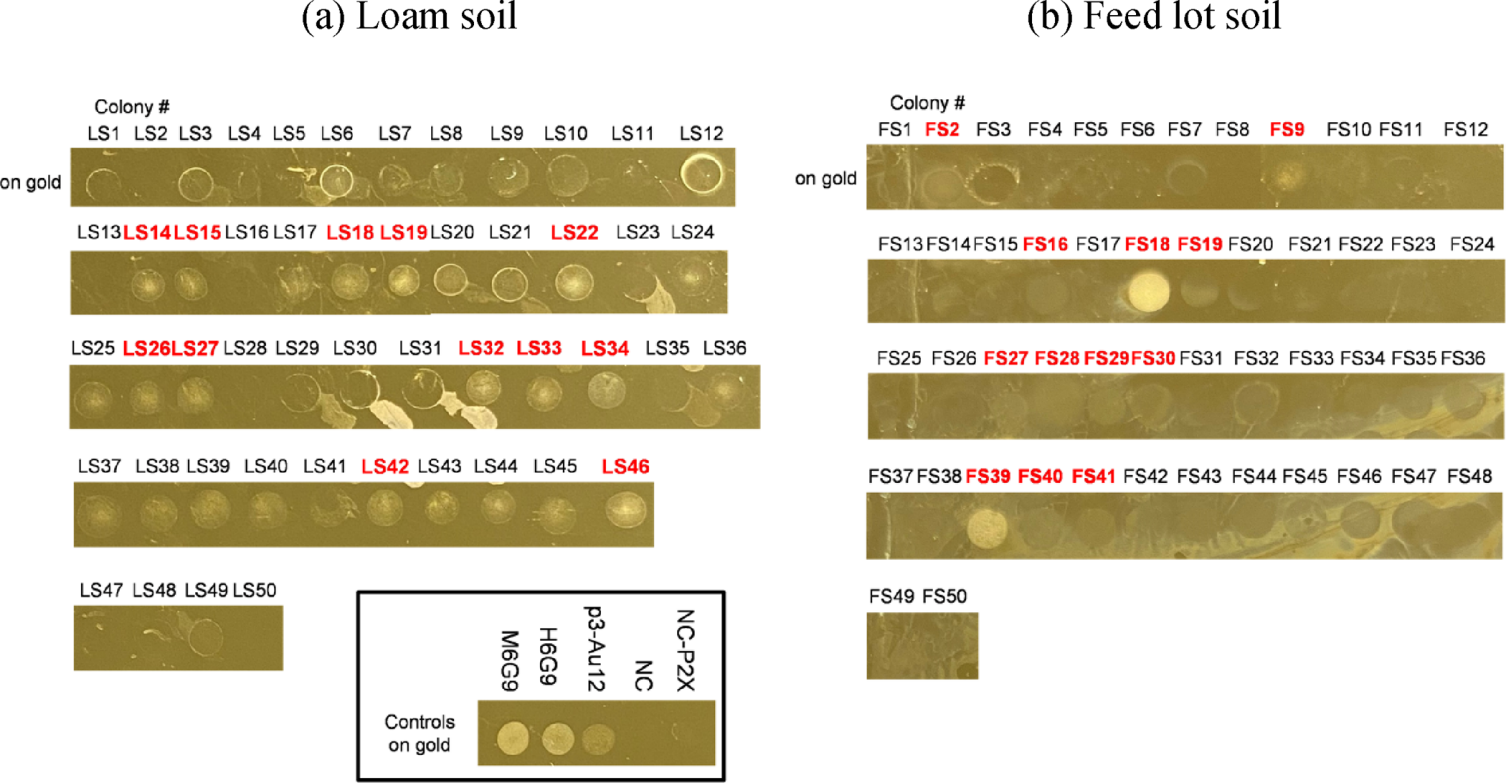
Surface binding spot assay of positive and negative controls and single colonies obtained from soil samples, (a) loam soil and (b) feed lot soil, using gold-coated magnetic nanobeads. Nanopore sequencing was conducted to identify the microorganisms highlighted with red color.

For each soil sample screening, 50 colonies were randomly picked from the agar plates and surface-binding spot assays conducted on the gold surface (Figure 6). The result of the surface-binding spot assay with the loam soil (Figure 6a) demonstrates that most isolated microorganisms were confirmed to show at least moderate binding to gold, comparable to the binding strength of the p3-Au12 moderate gold binding strain. The feedlot soil sample had a lower percentage of microbial isolates confirmed to show more than moderate binding (Figure 6b); however, there were a few strong binders (FS18 and FS39) to gold, showing comparable binding strength to that of M6G9 and H6G9 to gold. From each soil sample screening campaign, 12 microorganisms (from the 50 isolates confirmed for their binding strength) with moderate to strong binding to gold were randomly selected and nanopore sequencing conducted to identify these microorganisms (Table S6). In the case of loam soil, three different strains were found, with several of the strains being *Stenotrophomonas* sp. G4, while one strain was identified to be either *Enterobacter ludwigii* (but possibly *Kosakonia cowanii* or *Enterobacter cloacae comples* sp. FDA-CDC-AR_0132) and another strain identified to be *Stenotrophomonas maltophilia*. From the feedlot soil screening, all strains were *Bacillus* sp. strains. FS18 and FS39, which have strong binding to gold, were identified as *Terribacillus goriensis*. Thus, we successfully demonstrated that the developed platform can be employed to isolate environmental microorganisms that show strong material-binding properties. The degree of binding strength between the same strains such as FS2 and FS18 is different, and we assumed that their subspecies are different; however, the nanopore sequencing method has a limitation to identify it. The next step will be the performance of Illumina bacterial whole-genome sequencing to identify their subspecies and the surface peptide, which contributes to the specificity. Additionally, even the same subspecies can mutate to better interact with its environment, which can be an additional factor here. To understand if this is the case would require further analyses, such as whole-genome sequencing and/or proteomics, to understand if there are genetic differences or protein expression level differences in organisms of the same subspecies. We expect that the discovered microorganisms can be employed to develop new hybrid materials in the future. Considering that broad ranges of metals can be used to coat magnetic nanobeads, the developed microfluidic system and method are expected to have broad utility in varieties of applications.

## 3 Conclusions

In this study, we demonstrated the development of a novel biopanning process through the use of a magnetophoretic microfluidic platform to isolate microorganisms that can specifically bind to target materials of interest. Here, gold was the target material, so gold-coated magnetic nanobeads were used where microorganisms with high affinity to gold will bind to these nanobeads. The microfluidic platform allows continuous separation of these cells, where only magnetic nanobead-tagged microorganisms are separated into a collection outlet due to the applied magnetophoretic force, while non-binding or weakly binding cells can be removed through the waste outlet since in the absence of attached magnetic nanobeads they are unaffected by the applied magnetic force. This allows microorganisms with high affinity to target materials of interest to be enriched with high recovery, high purity, and high throughput. Using this platform, we successfully screened the eCPX 3.0 library that displays unconstrained 15-mer peptides on the bacterial cell surface and identified 100 isolates that bind to only gold or to both gold and ITO. We then characterized the correlation between specific peptides displayed on the surface of these cells and specific binding capabilities of these cells to gold and ITO. Additionally, the developed microfluidic platform was used to isolate soil microorganisms with high affinity to gold. This platform can be broadly exploited to screen other types of display libraries or microbial libraries against practically any material of interest that can be coated onto magnetic beads. This universal platform is expected to be utilized to identify peptides and new environmental microorganisms that show strong and specific binding to target materials of interest, which can then be utilized toward developing novel biological materials and hybrid organic–inorganic materials in the future.

## 4 Experimental Section/Methods

### 4.1 Fabrication of the Magnetophoretic Microfluidic Biopanning Device

The microfluidic device consists of a 0.7 mm-thick borosilicate glass substrate (1 by 3 inch^2^) on which inlaid ferromagnetic wires were formed within a microfluidic channel made of polydimethylsiloxane (PDMS). To create the inlaid ferromagnetic wires, a Ti/Cu/Cr seed layer for permalloy (Ni_0·8_Fe_0.2_) electroplating was deposited on the glass substrate using electron-beam evaporation. Photoresist (SU-82050, Kayaku Advanced Materials, Inc.) was spun and patterned to create a 50 μm-thick micromold for creating the ferromagnetic permalloy wires. Ferromagnetic wires of 50 μm thickness were then electroplated onto the bottom glass substrate. The 30 μm-thick ferromagnetic wire array was subsequently formed and inlaid in the bottom glass layer using a mechanical polishing technique. The angle of the wire is set to 5.7° in the direction of flow, the width and thickness of the wire are 50 and 30 μm, respectively, and the gap between the wires is 300 μm.

The microfluidic channel was fabricated using a conventional soft lithography process. First, the master mold made of a 50 μm-thick layer of SU-82050 photoresist (MicroChem, USA) patterned on a 3-inch silicon wafer was created. This SU-8 master mold was coated with tridecafluoro-1,1,2,2-tetrahydrooctyl-1-trichlorosilane (United Chemical Technologies, Inc., Bristol, Pennsylvania) to facilitate the release of the replicated PDMS layer (10:1 mixture, Sylgard 184, Dow Corning, Inc., Michigan). After oxygen plasma treatment of both the glass substrate with inlaid ferromagnetic wire and the PDMS replica, they were aligned and bonded together for 24 h at 80 °C. The width of each inlet is 500 μm, the length of the microchannel where the magnetophoretic separation was performed is 3 cm, and the widths of outlets #1 and #2 are 200 and 800 μm, respectively.

### 4.2 Cell Preparation and Magnetic Nanobead Binding

Unless otherwise stated, the bacterial strains were grown in 5 mL of lysogeny broth Miller (LB) supplemented with 25 μg mL^–1^ of chloramphenicol (LB-Cm_25_) media at 37 °C, with shaking at 225 rpm overnight. Of the overnight cultured bacterial strains, 60 μL was taken and was resuspended in 3 mL of LB-Cm_25_ media (1:50 dilution) and incubated at 37 °C, with shaking at 225 rpm for 2 h. When the range of optical density at 600 nm (OD_600_) was between 0.4 and 0.6, 30 μL of 4% w/v l-arabinose (Millipore Sigma, 0.04% final concentration) was added to the cell cultures to induce the eCPX display scaffold for peptide display on the cell surface. Cells were incubated at 37 °C, with shaking at 225 rpm for an additional hour. The cell cultures were chilled on ice for 10 min to stop induction and centrifuged at 3000 × *g* for 10 min. The supernatant was removed, and the cell pellets were resuspended in 4 mL of PBS solution. The core of the magnetic nanobead is 10 nm of Fe_3_O_4_, which is covered with 5 nm of gold layer, and the gold layer is capped with citrate, thus the overall diameter of the magnetic nanobeads is 20 nm (A1M1-10-5-CIT-DIH-2.5-1, Nanoparts). 100 μL of magnetic nanobeads solution (10^14^ magnetic nanobeads mL^–1^) was added to the cells suspended in PBS solution, and then the mixture was placed on a roller shaker to avoid the sedimentation of magnetic nanobeads and incubated at room temperature for an hour.

### 4.3 Device Characterization

Two *E. coli* strains with known material binding properties (A3 and A68 (Table S1)) were used to characterize the developed microfluidic platform. These strains were isolated from a previous study.^24^ A68 showed strong binding to gold and A3 original cells showed very weak binding to gold, compared to the degree of binding strength of NC-P2X (negative control) against gold. After eCPX expression was performed for these strains by induction with l-arabinose, approximately 5 × 10^8^ cells of each of the induced strains suspended in 1 mL of PBS solution were incubated with 20 μL (approximately 2 × 10^13^) of magnetic nanobeads. A magnet (NdFeB, Grade N42, 3/2″ length × 1/2″ width × 1/16″ thick, BX881, K&J Magnetics, Inc., USA) was placed underneath the microfluidic device (with an external magnetic flux of 1.3 T). The flow rate for sample and buffer injection was set to be 2 mL h^–1^, and all debris, non-specific binder cells, and weak binder cells were flown through outlet #2 at 3.2 mL h^–1^ of withdrawal flow rate. Each bacterial strain was injected into a separate replicate device, and cells bound to gold-coated magnetic nanobeads were separated into outlet #1 and collected for 10 min. Serial dilution of each collection (total volume: 1 mL, 10-fold dilutions from 10 to 10^5^) was followed by a 10 μL spot plating on LB-Cm_25_ agar for colony counting to compare the binding level across samples.

### 4.4 Microfluidic Biopanning

The eCPX 3.0 bacterial display library (approximately 10^11^ cells) was cultured, induced, and mixed with magnetic nanobeads, as described above for device characterization. The library was suspended in 4 mL of PBS buffer and collected in a 5 mL syringe. The flow rate setting for the two inlets and outlet #2 was the same as described above, and the cells separated into outlet #1 were collected for 2 h. A total of four sorting rounds were implemented with the same conditions for rounds 1 through 4. The collected cells were cultured in 500 mL of terrific broth (TB) media containing 25 μg mL^–1^ chloramphenicol and 0.2% glucose (TB-Cm_25_ glucose) and cultured at 37 °C with shaking at 225 rpm overnight. Based on the assumption that an OD_600_ of 1.0 has an *E. coli* concentration of 10^9^ colony-forming units (CFU) mL^–1^, about 10^11^ cells were collected, centrifuged, and then used for the next sorting round.

### 4.5 Off-Chip Surface-Binding Spot Assay

To analyze the affinity of the sorted peptide library cells, 50 colonies (original cells) were randomly selected from each of round 4 and round 1 of library sorting. The plasmids of the original colonies were extracted (D4210, Zymo Research), followed by transformation into MC1061 *E. coli* cells (ATCC 53338) made chemically competent using the Mix and Go! Transformation Kit (T3001, Zymo Research), which we call “Z-competent cells”. The Z-competent cells of each colony were cultured and induced as described above. All cells notably grew at about the same rate and were induced at similar times. The induced Z-competent cells of each colony were used to conduct the surface-binding spot assay on gold and ITO layers, where 2 μL of cell solution was dispensed and incubated for 30 min on the material surface at room temperature, stationary. Non-binders were pre-washed by slowly pouring 5 mL of 1% Tween 20 (PBS-T) to the material surface away from the spots in order to avoid smearing the spots, and then they were washed by moving the substrate to 20 mL of PBS-T in a 50 mL conical tube and shaking horizontally at 150 rpm for 20 min on a rotating platform, followed by taking images of the cells remaining on the gold and ITO layers.

### 4.6 Off-Chip Flow Cytometry and DNA Sequencing Assessment

To quantify the P2X peptide display, and therefore eCPX scaffold expression, using flow cytometry (BD Accuri C6 Cytometer), 5 μL of each cell culture was suspended in 25 μL of PBS containing 150 nM YPet-Mona (ex/em: 517/530 nm), as previously described.^41^ The flow cytometer measurement was considered complete when the total number of analyzed cells reached 10,000. Data was analyzed using FlowJo software (TreeStar, San Carlos, California, USA). The average YPet-Mona fluorescence intensity area (FITC-A) of each colony was analyzed and presented as a histogram. The bulk of the population of the negative and positive controls within the area of autofluorescence on the plot was determined by gating or confining. The median fluorescence intensity (MFI) was also calculated. All peptide sequences were determined by isolating single colonies and DNA sequencing directly from the colonies using the pBAD Forward universal primer (Genewiz), followed by translation of the DNA sequence. The macro file provided in Sarkes et al. was used to simplify this analysis.^41^

### 4.7 NGS of Round 1 and Round 4 Sorts

For each of the round 1 and round 4 sorts, plasmids were isolated from freezer stocks using the ZymoPURE II Plasmid Miniprep Kit (Zymo Research; Cat. No. D4201). A sequencing library was prepared according to the Illumina 16S Metagenomic Sequencing Library Preparation protocol (Illumina; Part # 15044223 Rev. B) using custom forward (AGTTCTGGCTTTCACCGCAG) and reverse (CCGTAGTACTGGTTTTTGTTGTAGTC) primers corresponding to eCPX 3.0 scaffold regions adjacent to the peptide insert (primers also included standard Illumina platform adapter overhangs (not shown)). Samples from round 1 and round 4 sorts were multiplexed using the Nextera XT Index Kit V2, pooled, and 2 × 151 bp sequenced on an Illumina iSeq 100 (Illumina, San Diego, California, USA).

### 4.8 Analysis of Frequency of Amino Acid Residues

Processing of NGS files is performed with a combination of Linux commands, awk, and python and associated libraries Biopython, NumPy, and matplotlib. Display peptides in the eCPX system are bounded by a nucleotide precursor sequence (GGCCAGTCTGGCCAG in the wild type, which translates to GQSGQ) and a postcursor sequence (GGCTCGAGC in the wild type, which translates to GSS). Strings containing the [precursor]–[display peptide]–[postcursor] nucleotide sequences are stripped from the original NGS fastq file and converted to protein sequence with Biopython. Sequences containing stop codons, frame shifts, blank inserts, and unrecognized residues are removed, and the precursor and postcursor strings stripped. This leaves a list of “valid” display peptide sequences. Only valid display peptide sequences of 15 amino acids in length are considered, although some statistics of display peptide length within the library are included in the supplemental information for the interested readers (Table S4). Total occurrence of all 20 naturally occurring amino acids in the list of valid 15-mer sequences is accumulated and normalized by the total number of residues within the list (to provide a similar scale for ease of comparison between the eCPX 3.0 library and gold binding sequences).

### 4.9 Soil Sample Preparation

The collected soil sample from pristine ecosystem and agricultural animal use was stored at 4 °C. For soil dispersion, 4 g of soil was added to PBS supplemented with 0.5% Tween 20, and then the mixture was blended at a maximum speed for 3 min at 1 min intervals with 1 min incubation on ice. For soil density gradient centrifugation, 80% Nycodenz cushion is made by adding 80 g of Nycodenz (AXS-1002424, Cosmo Bio) in 100 mL ultrapure water. 18 mL of Nycodenz cushions was added in a centrifuge tube, and then 20 mL of the blended soil mixture was slowly loaded onto the top of the Nycodenz cushions. The tube was centrifuged at 15,000 × *g* for 40 min at 4 °C. The layer containing cells was transferred to a new tube and 20 mL of PBS was added, and then the mixture with PBS was filtered by using a strainer (pore size: 30 μm) and centrifuged at 15,000 × *g* for 40 min at 4 °C. The supernatant was discarded, and the cell pellet was resuspended in 5 mL of PBS and then stored at 4 °C before use.

### 4.10 Nanopore Sequencing of Microorganisms Isolated from Soil Samples

DNA from microorganisms, isolated from two separate soil samples (loam soil and feedlot soil), was extracted using the E.Z.N.A. Bacterial DNA Kit (Omega Bio-tek; Cat. No. D3350-01) and prepared for sequencing using the Rapid Barcoding Kit (Oxford Nanopore Technologies; SQK-RBK004). Twelve samples were pooled and then sequenced for approximately 24 h on the GridION (Oxford Nanopore Technologies; Oxford, UK). Kraken 2 (https://github.com/DerrickWood/kraken2/wiki) was used to perform taxonomic classification on the reads obtained from sequencing for each of the samples.

## Supporting Information Available

Image of the fabricated microdevice; microscopy images of the microchannel; COMSOL simulation; sequences of peptides displayed on the surface of *E. coli*; analysis of separation efficiency; analysis of P2X peptide expression; statistics for eCPX3.0 library and round 4 and round 1 of library sorting against gold; distribution of length of valid display peptide; raw count of amino acid occurrence along the length of valid 15-mers; sequence and frequency of occurrence of top 100 most frequent sequences; heat map of co-occurrence of amino acids; and identification of environmental microbes (PDF)
