# Supplementary material for: Identification of Microorganisms that Bind Specifically to Target Materials of Interest Using a Magnetophoretic Microfluidic Platform
Source: ACS Appl Mater Interfaces. 2023 Feb 27;15(9):11391–402. doi: 10.1021/acsami.2c15192 (PMC10848205; doi:10.1021/acsami.2c15192)
Supplement: Supplementary file 1 — am2c15192_si_001.pdf [file am2c15192_si_001.pdf]

Supporting Information

Identification of Microorganisms that Bind Specifically to Target Materials of Interest using a Magnetophoretic Microfluidic Platform

*Song-I Han[1], Deborah A. Sarkes[2], Margaret M. Hurley[2], Rebecca Renberg[2], Can Huang[1], Yuwen Li[1], Justin P. Jahnke[2], James J. Sumner[2], Dimitra N. Stratis-Cullum[2], and Arum Han[1,3,4]**

Song-I Han, Can Huang, and Yuwen Li

[1] Department of Electrical and Computer Engineering

Texas A&M University

College Station, Texas 77843, USA

Deborah A. Sarkes, Margaret M. Hurley, Rebecca L. Renberg, Justin P. Jahnke, James J. Sumner, and Dimitra N. Stratis-Cullum

[2] Biotechnology Branch

U.S. Army Combat Capabilities Development Command (DEVCOM)

Army Research Laboratory (ARL)

Adelphi, Maryland 20783, USA

Arum Han

[1] Department of Electrical and Computer Engineering

[3] Department of Biomedical Engineering

[4] Department of Chemical Engineering

Texas A&M University

College Station, Texas 77843, USA

E-mail: arum.han@ece.tamu.edu

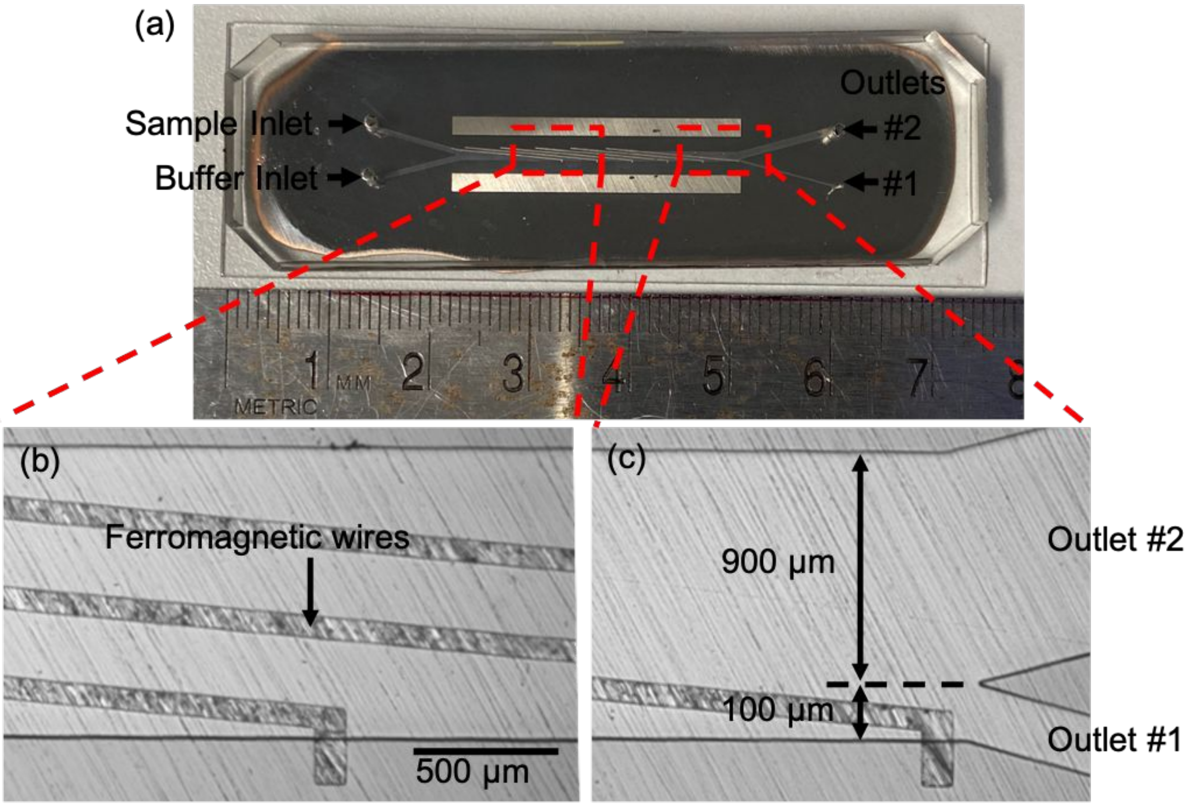

Figure S1. (a) Fabricated microdevice. Microscopy images of (b) the middle of the microchannel and (c) the end of the microchannel where the microorganisms with high affinity against target material of interest are separated into outlet #1, while unbound magnetic nanobeads and unbound and weakly bound cells are separted into outlet #2.

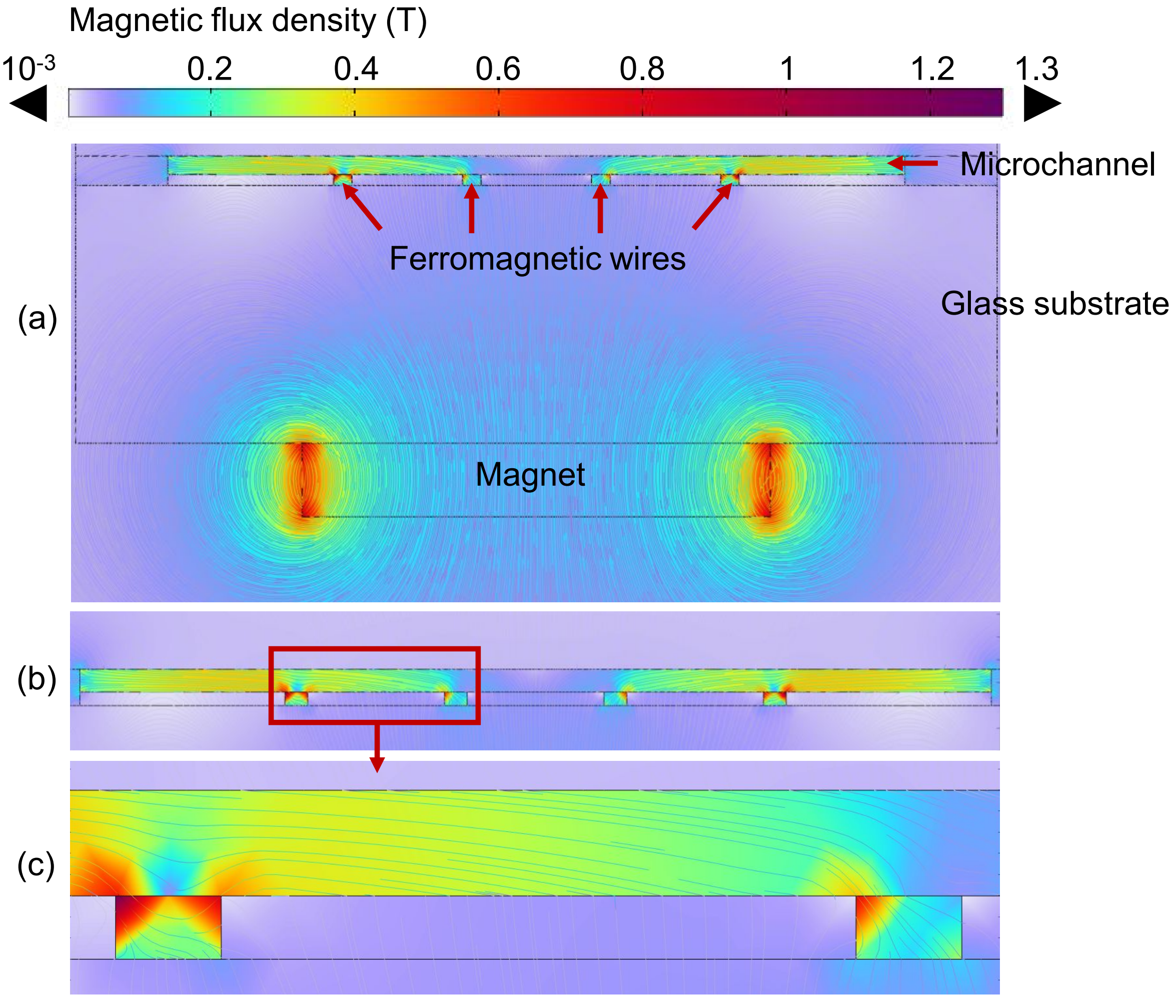

Figure S2. COMSOL simulation result showing the magnetic flux density (T) across the microchannel (COMSOL Multiphysics® 5.5). (a) The device consists of a glass substrate on which ferromagnetic wires were deposited, and a microfluidic channel made of PDMS. A magnet (Br max: 1.3T) was placed underneath the device and generated magnetic fields. (b and c) An enlarged view of the microchannel. The egde of the wire, close to the edge of the magnet, has the strongest megnetic field (1T), resulting in about 0.6 T magnetic field generation in the microchannel.

Table S1. Sequences of peptides displayed on the surface of *E. coli*.

E. coli variants	Peptide sequences
NC-P2X[a, d]	GQSGQ and HISQWKPKVPNREDKYKK
A3[b, d]	GQSGQ-RVAYPYCCFMASPGL and HISQWKPKVPNREDKYKK
A68[b, d]	GQSGQ-SLCDSNNFAHTGVLR and HISQWKPKVPNREDKYKK
P2X[c]	HISQWKPKVPNREDKYKK
NC[a,e]	GTSGQ
p3-Au12[b,e]	GTSGQ-LKAHLPPSRLPS
M6G9[b,e]	GTSGQ-MMMGGGMGGGMGGGM
H6G9[b,e]	GTSGQ-HHHGGGHGGGHGGGH

[a] These strains (NC-P2X and NC) are known negative controls that do not bind to gold.

[b] These strains (A3, A68, p3-Au12, M6G9, and H6G9) are known moderate to strong binders to gold, and are used here as positive controls. Affinity of these strains to gold varies as described in the text.

[c] The P2X peptide at the C-terminus was previously shown to have weak affinity to gold. This strain is also a positive control for peptide expression level.

[d] These strains (NC-P2X, A3, and A68) were previously isolated from the eCPX 3.0 library and display GQSGQ at the N-terminus before peptide insertion and P2X (HISQWKPKVPNREDKYKK) at the C-terminus after peptide insertion.

[e] These strains (NC, p3-Au12, M6G9, and H6G9) display GTSGQ at the N-terminus before peptide insertion but lack the P2X peptide at the C-terminus.

Table S2. Analysis of separation efficiency based on the number of colonies formed on LB-Cm_{25} agar plates after the separation through the microdevice.

Strain	Initial cells injected (Cells mL^{-1})	Dilution rate	Counted cells on LB-Cm25 agar (four replicas)	Average (Standard deviation) of counted cells on LB-Cm25 agar	Separation efficiency (%)
A68	3.3×10^8	10^4	226/231/230/221	227±4.5	69
A3	3.3×10^8	10^4	100/82/114/92	97±13.5	29

Table S3. Analysis of P2X peptide expression (shown as % binding to 150 nM YPet-Mona) for the Z-competent cells with the same plasmid transformed from the original cells isolated from the eCPX 3.0 library as well as confirmation of their amino acid sequences (AA sequences). (MFI: mean fluorescence intensity)

Gold							
**Colony #**	**AA Sequences**	**YPet-Mona (%)**	**MFI**	**Colony #**	**AA Sequences**	**YPet-Mona (%)**	**MFI**
Z-G-1	VAGQSGRCSCSGLQG	90.6	10638	Z-G-26	HYVTKGSPAHSMCNA	91.5	19015
Z-G-2	DRKSHNHYAASLAPV	96.1	2912	Z-G-27	SSSKVQHHNVPRPDS	96.8	4336
Z-G-3	SSCSRSPAGLTMLRT	95.8	14544	Z-G-28	YIVRSPSPEGRGCIR	92.1	10638
Z-G-4	AVCRKPYSEQVVVRP	95.6	14426	Z-G-29	IGRGLPHSLSRLTLT	91.4	6351
Z-G-5	PRHQSQSYTSNLQLL	94.2	10009	Z-G-30	RASDPASFRLDSKIP	94.5	5163
Z-G-6	CHYAVHPVPGNPSLR	90.4	19563	Z-G-31	SKLTCRSSVLCYTFV	93.3	6198
Z-G-7	YPAKSARLPSCITAH	91.0	51852	Z-G-32	LKFTHHHCNAAVD	93.3	6508
Z-G-8	NRVHLISDRTCRSEL	92.6	15647	Z-G-33	HLTVTHQSYSSSWNS	92.7	11261
Z-G-9	CHLQSFSAGQEKMQL	90.5	4897	Z-G-34	ASRHHVQMGFWSHTG	90.4	4571
Z-G-10	FNGTPSLPRPQSSC	90.3	15270	Z-G-35	QAQVQSKLQPTGYRH	89.9	7030
Z-G-11	NPRHRPPFAAHTEVL	92.1	6668	Z-G-36	DSPGSGIHCLHHSHS	92.9	9729
Z-G-12	GCRVHELSHTPTRAK	92.7	6833	Z-G-37	VSLHLSHHCQSSSRR	94.6	2200
Z-G-13	PRQGSLLRCGNYDTH	92.5	15520	Z-G-38	SSQRLLLAARHGSFL	92.4	4998
Z-G-14	TVICCFPLRHENHAL	94.0	10638	Z-G-39	GQINLSRSHRPLSLD	94.1	3641
Z-G-15	HLLVGTRLHGSGPSL	90.0	10595	Z-G-40	QSGQLCSGEYKRSRI	91.4	3885
Z-G-16	SVAGQSGRTSCFSTH	86.7	11632	Z-G-41	TVGGSLHSIKGLPKS	94.6	3731
Z-G-17	AHVGSSTRPQTQSYQ	91.6	7845	Z-G-42	CGLHLQNRPTLMLPG	88.7	7941
Z-G-18	GVNRHQLHTKHPQDN	95.9	10638	Z-G-43	KSSKPVGSHVPIKSN	91.2	3807
Z-G-19	HITRYNCNGDFRHIG	96.2	9808	Z-G-44	HSYCATYQQRFTSM	91.9	9689
Z-G-20	ATHHANFRPDSVWLL	94.7	7116	Z-G-45	FGHLSCLNLPRPSKP	93.4	4799
Z-G-21	VCSLTQGSKMHHACY	93.9	7001	Z-G-46	SRHDRELSYSPGFGR	95.0	6274
Z-G-22	KCRPISYSAGISAHQ	96.6	14963	Z-G-47	TFISMADKRLHAQSF	95.4	2610
Z-G-23	NPRPCNVGKPLATLS	93.2	20292	Z-G-48	IPASKHSGPHCALIS	94.0	6833
Z-G-24	#EMPTY	95.7	6778	Z-G-49	IHRVPLLHVMQX	90.5	3854
Z-G-25	GGSRCMPLYSSSSIK	91.7	28082	Z-G-50	IARSSMVHLHVNELR	89.3	3454

Gold							
Colony #	AA Sequences	YPet-Mona (%)	MFI	Colony #	AA Sequences	YPet-Mona (%)	MFI
Z-G-51	FSDLLTSQLQMQNCR	96.2	4497	Z-G-76	DFVDXXRYYFRTLGS	2.29	216
Z-G-52	IPRAGRSSLPPHGHA	92.8	4703	Z-G-77	RYMNNSPSRALASYV	96.3	4917
Z-G-53	NVDGFSIFHKAPNYF	92.6	3440	Z-G-78	#EMPTY	96.4	4515
Z-G-54	ALCKTASCETSTHTG	91.8	5377	Z-G-79	ISMHGVTQRGGLGHI	89.4	5623
Z-G-55	CDRNDVRVKTPSSSV	86.6	5142	Z-G-80	SWLIRLSSLLDISIP	94.5	1711
Z-G-56	DTRLSNKLTRIGKQS	92.7	5142	Z-G-81	SIISKCASKFRFSRS	91.0	1413
Z-G-57	#EMPTY	82.2	6403	Z-G-82	TSRCPPCRSPGRTLH	96.8	2320
Z-G-58	EHCRATHNGALFPDL	90.6	4917	Z-G-83	SAVYHNAFIRSSNLT	95.5	4646
Z-G-59	#EMPTY	94.3	6098	Z-G-84	TSVAGQSGRRGGLLR	2.17	228
Z-G-60	LRTLPSLLASHAGRA	94.9	7381	Z-G-85	HSLVQPTQEAMRKAR	96.7	3997
Z-G-61	RASPKLEYKTSVPSW	94.2	4283	Z-G-86	LRPVKTGHETD	95.4	3184
Z-G-62	LKDYGFKCINNQTRG	93.7	3539	Z-G-87	TTSASCRANCLGMTS	95.8	3525
Z-G-63	SSQFVRNALPHGSKM	92.9	5377	Z-G-88	SGGLHTLHMRVKGRT	97.6	5465
Z-G-64	KHLPRFR	95.0	1901	Z-G-89	LLENLTRWGIPVRNS	97.0	3276
Z-G-65	INLF*CRYFYAFPPX	2.56	216	Z-G-90	RFESLMRGSIVHNWY	94.3	3761
Z-G-66	SACKLCAFHEAHHHS	95.4	9228	Z-G-91	AEQICSRLVRPGPAA	96.8	3823
Z-G-67	SHCTGSQHAPPTWSY	87.4	11871	Z-G-92	HSNRFAGPTS	96.6	3885
Z-G-68	VIG*ANVKCSPVATR	6.10	280	Z-G-93	IRFHLSPNVTSMGSF	95.9	2445
Z-G-69	RSLPVTQYMPRGFPP	93.7	9729	Z-G-94	#EMPTY	97.0	4063
Z-G-70	#EMPTY	92.3	4897	Z-G-95	RSHSVSRGTSSCGSL	96.7	5903
Z-G-71	SRLAVLAKQCKVHRV	91.5	3949	Z-G-96	SVCKPRSSVDTPSHG	91.8	3033
Z-G-72	LNLLYSTYQNSHTHS	92.0	2774	Z-G-97	CASYATSCFGKSCAR	96.5	4479
Z-G-73	RHSYDQIFGTMPRNQ	93.5	3197	Z-G-98	VLFWTSLCPXGYSAE	88.5	2012
Z-G-74	YMYDFWVRRGYQGLC	82.1	2165	Z-G-99	RHRPFPITTTGSRVL	96.0	3468
Z-G-75	DSRICFLSTLLGNQH	92.3	1811	Z-G-100	#EMPTY	93.9	4703

Table S4. Statistics for eCPX3.0 library and round 4 and round 1 of library sorting against gold. Total number of valid sequences, all lengths, represents number of sequences in which all of the following are true: (1) correct precursor sequence before display peptide sequence, (2) correct postcursor sequence, (3) no stop codons, (4) no frame shifts, (5) no blank inserts, and (6) no unrecognized amino acids. Total number of valid 15-mer peptides represents the count of valid sequences which are 15 residues in length. Number of unique 15-mer peptides counts unique valid 15-mer sequences. The final column provides the number of occurrences of the most frequently appearing sequence in each library.

	Total valid, all lengths	Total number of valid 15-mer peptides	Number of unique 15-mer peptides	Count of most frequently occurring sequence in library
eCPX 3.0 library	90781520	86902028	81150364	416
Round 4	2209883	2070355	1893542	1084
Round 1	1918970	1800643	1669002	8

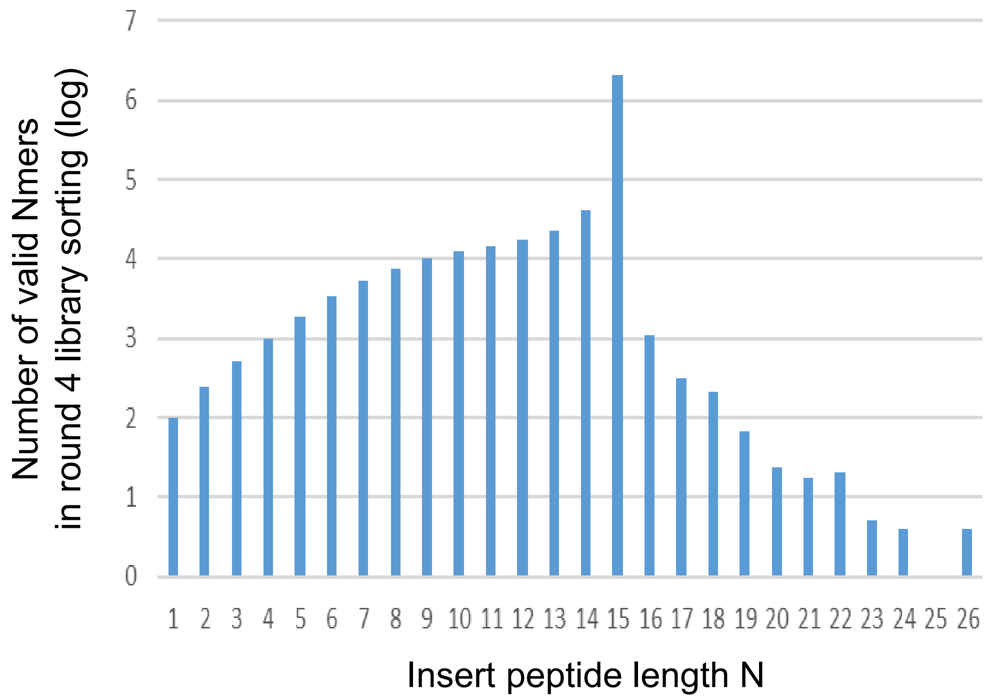

Figure S3. Distribution of length of valid (see definition above in Table S3; all 6 conditions must be met) display peptide inserts in round 4 library sorting using gold-coated magnetic beads.

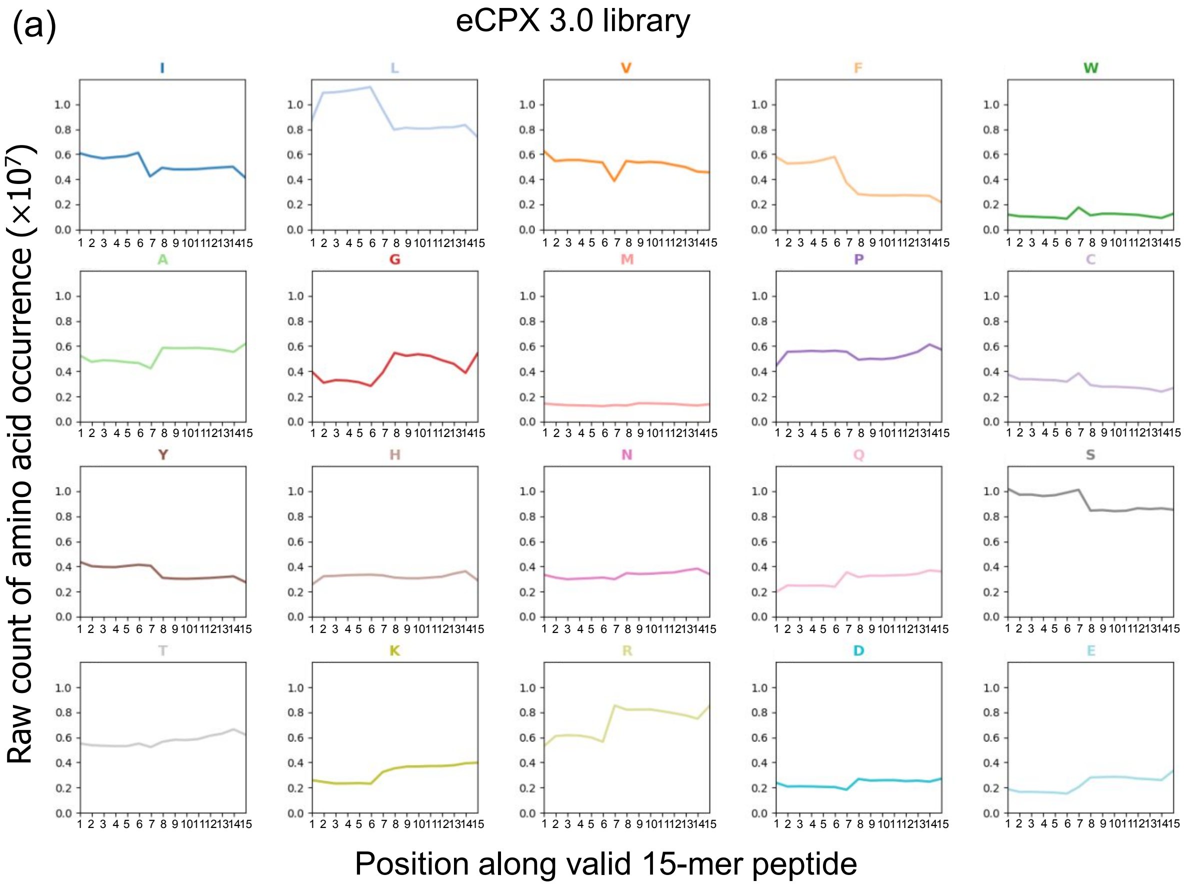

(a)
eCPX 3.0 library
I
L
V
F
W
A
G
M
P
C
Y
H
N
Q
S
T
K
R
D
E
Raw count of amino acid occurrence (×10^7)
Position along valid 15-mer peptide

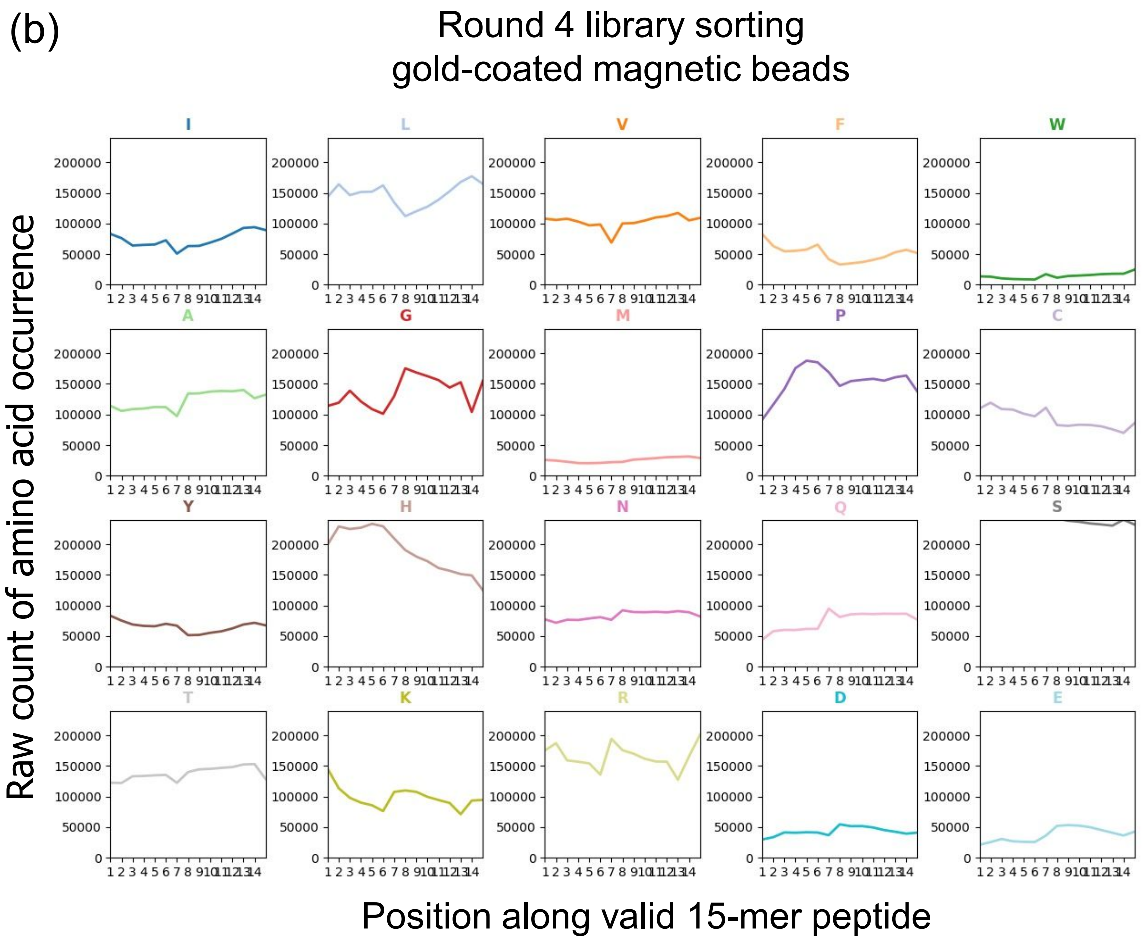

Figure S4. Raw count of amino acid occurrence along the length of valid 15-mers in (a) eCPX3.0 library and (b) round 4 library sorting using gold-coated magnetic beads. Single letter amino acid codes are used here above each graph (e.g. I = isoleucine).

Table S5. Sequence and frequency of occurrence of top 100 most frequent sequences in the eCPX 3.0 library and round 4 and round 1 of library sorting against gold.

Top	eCPX 3.0 library		Round 4		Round 1	
	Frequency	AA sequence	Frequency	AA sequence	Frequency	AA sequence
1	416	RLFCTNKTANKKSKT	1084	YYHRDGKGEYKARHE	8	HDNLHASREDCGVGT
2	340	PTLRFHSTLPSDNVN	918	TIHRPGGSGANSALK	6	YPFLCQSVTRIPTPS
3	308	RSLSCFRAQTLSPRQ	350	LGTPASSHCGRSHVC	6	YNCRIHNEKVAKKNG
4	289	RAYLRKGPSRWTLSL	312	NILFKTKSNCGRNID	6	YAHYSASPYLYYQDS
5	273	YSNIASSCWLVRIGR	306	HIGATLSGPSLFSTY	6	VRYVQAFQAIRPNLR
6	268	RFSNYACLVMPPPLL	291	RQSTRVSSTALYHSS	6	VLSYPQCDKARVALS
7	266	CLRVPFGEDPTKHQP	284	LNSTASNGSHTNYGK	6	GLICLYSCVLHQKPR
8	259	VRLCPSPTIIVNTIV	261	LSPHWCRSLSHCFQS	6	GINIPTCDLCTPMSG
9	255	LYFTHGHVTSFVASS	253	LHQHSRFGGEGRQSY	6	FHHAPCSYAPGLLKD
10	246	RFWCVSFYVANYFAT	242	LHLPVSKHGRTINMD	5	YTRLHLSANRNIHSE
11	242	PCVLKYCRQITRQAN	237	PGHSRLKTCPYIHSI	5	YTDTLFGLFCVGCAP
12	240	VKPSEKKCYLSQRLR	233	SWPHCTSEGRSACHV	5	YPRTELGKSIRNNVS
13	224	LLTSTFWQSKQLDNS	204	RKSLGSANKPSYVYF	5	YALHFPTEDRYRMPN
14	222	ATLYLGLYFLVPQDT	196	RSSHYHGKEACHLQT	5	WPGHATYDRVFKCCS
15	217	YLRSITHESTRTRAD	186	LLSVSPQRSKPLHAL	5	VYAFPGRKCGPDDVC
16	214	SCFYLRSTGSSPWAK	182	LGQTCKHSINRPQTP	5	VRPTLVSCTSANRPS
17	214	ALKPSRRHLHLFEHR	169	IRAAPKLSSHHVKTG	5	VRHHCSGIPSPDDVI
18	213	CNSFGSESLTPSWHK	162	HGLGSHNRVPAQGRF	5	VLLPFSQSHVHSTLS
19	205	TRVHSCFSLEVPDLE	157	SACMFQSRGAKSPAL	5	TSLSPDFFVLPYLAP
20	204	FYKQIQLGSRKNLPP	157	LSGIRNPACTTISSR	5	TRDLLLMSDSTSRIP
21	203	VVARLACYCDRMFSC	152	PKLSFNGRPQTTPYH	5	TPVHEFHKYLPLTVS
22	202	FYVCHARKELYRHTT	151	NSPHNCRNISVIHKY	5	SWPAHITGRSQKLLT
23	202	FKRYVSQITLRFLQN	138	HHIKLPREPGQITLP	5	SVSTQFLRIPTMQRE
24	201	WPLVRFNLSTHSTPP	134	YSVGFPHSRTSTLQH	5	STVSPGHHCHVPPES
25	200	RSSFVMFCHIRRVNR	128	SISSAHSMFQHRTRV	5	SIPFRVPQLLFGPRY
26	200	RQLIYQSTSYAARQN	120	RTYSASEESQHSNYK	5	SDRIHLRRGTCHTKP
27	197	FLLSFTSVGLSRRSI	119	GLQTTLISRSNKCCV	5	RYHQHVSKGHRPSSL
28	197	DGKPLSTRRWTASAR	118	SNSRVTHSGLGTEVV	5	RIPGYAPTHQHVADK
29	194	QYIFAFGTAYSREAR	110	GSHCTRLDLPACYRN	5	RFPGEASHNPGRERM
30	194	KCARDHWSKGSQKPL	109	PASGHYKHRTICTPA	5	QVNLRELRSSRHSQG
31	192	YSFILESSIRDISEV	102	TAHYIDRNSQPFQVN	5	QKLTDVYKKAPLVLT
32	192	VASNGVCYFRTERTL	99	RHQILLSQQIQPRTS	5	QCSPFSRTATMCGLQ
33	192	SVLFLPCKTNPSQSN	98	RVSSCYTSAGLSHEG	5	PSVQITNADKYGQCH
34	188	SWYISPSRAASMRDL	98	PGSKRCSRHIHLLLP	5	PSRDKLLKVGNSHSR
35	186	YAAGGIILSIDRADR	98	CAKLPEINRRTPLLG	5	PSFCKSYPLTPHVRP
36	182	LQICCYIRVEVSSQD	97	HYFRVHQVLPGSQVN	5	PRHDKHLTKVDLRST
37	179	PSRNRASLASNLVNA	90	YSHTTSSISGVQRYP	5	PLCQSTPHVGIKPPG
38	177	LYKFRYKNSLHTSIE	89	SQRLFDCKSSYMPYT	5	PAQKAYSTCALTTRH
39	174	VSRHLISRHTLYKLD	89	NLPPLKLHAHPPGSS	5	NGPTVSVNNRSCRLP
40	173	FDIRSFQSSHDRPNS	85	CHNRFLRTAKSMVPR	5	LSSHRLLRPQPIGAP
41	169	EPCFTGSERGNLSPC	84	KPCLGRSNARSNILQ	5	LPVEAARHERYPRTN
42	167	LCLVGFCTGLRYCRI	82	LSGPLSHRHHLIYPI	5	LIVPIVSTTPVYHGI
43	162	WHNPRRLILCARNRG	81	VTKLHRQDLVPLAGC	5	LIKPILCQFSRHKIL
44	161	SLQDISRWGQQTPGA	78	PKRHSTEKYQRNIIF	5	LFRHFSKHTTHSIVV
45	160	IPFSFTVPWSKGRTP	75	LSDHSLKSARAGLEC	5	KPRDYTLLTHSPGHQ
46	156	IFSTPAHACLGRSIT	75	HACFNMRDYLVRSPK	5	KMPANSVPTLKAGLR
47	153	FSYYLLFGTYTLCYG	75	GSTKANRSDTLYGPG	5	KLPIPELSAVGIFGR
48	152	MSVSVLQNDVHNGPS	74	HPCPFNVQDNRRWVQ	5	KIPVPSVVTDNCSRG
49	152	DVPKPCFSAKAVVRF	71	RCPDLISTHTPRGLS	5	IRRLIRLNGGIATFE
50	149	VIHFTKLQWSHRANV	70	QSMRAIKENSVRHCT	5	HIEYLSAKKSLAHSI
51	148	LQLPGSERQTTYIAQ	70	IRQYCNHRVGSQGPS	5	HCLCRGQLCLSAHSC
52	148	GFHYLNWSVHPVGEQ	69	QLAISRCGTKNHHLK	5	GYSGSDSLRLRMGSS
53	147	HIPRALSISANARAF	69	LSHSPIANDRHHASD	5	GSLVLLNRHTTCLAT
54	147	GCRLVAQASIFLGDY	68	RGRLTNPQSEVIHII	5	GLIVPRSAHMLTFTH
55	147	DCSLGARKNALDRPP	67	GATLRASTGRSSHVE	5	GCMYTNRRCQPTWHV

Top	eCPX 3.0 library		Round 4		Round 1	
	Frequency	AA sequence	Frequency	AA sequence	Frequency	AA sequence
56	147	DCIEHLYLEKTVSNS	66	SSFGVRHLQPRVSGQ	5	FTDFFSLNHVRSPYV
57	146	RSSAAVSLTPGSLQE	64	ACSISFFRSHCDLSN	5	FNSDLPLMMLLSCLP
58	146	LCVYVCWRKLPGCNT	62	NGAAATRVGSRSDSA	5	ETACWPTTNTDKTRP
59	146	CILLFYPHCKYTPHT	61	THVSIERSHAWSHST	5	ENHTLSHHPLRVSFA
60	145	YSRNYPEHIMLNNIS	61	GVRSSLHHTVPRFPE	5	EIMTRRAGVDRYMPI
61	144	VDWSRSIEPKGNSIV	61	FAQKPGCALKGNIDR	5	ECHRAATTLRRGPTI
62	141	KCVSLRSCGLEDAFT	59	NETCLSSRNSPFISN	5	DCSFTLPTQSYCEHR
63	141	CMYPKLAHPRGILLD	58	VRLSGGSPGHCTVIR	5	DALTRSLSRPCRSHE
64	140	SSSHFALASVQAFIL	58	ICPQSLPSQNLMSHV	5	CIMRAGVDTTQNKNY
65	140	GRGNLRHSWTHRITL	55	YHCPKPNAVSSIYYK	5	CGRQEFQIQVPFNSL
66	140	CICKARDQIACSMRI	53	LSASVPSLSHGHAIP	5	CDLQATQSKSWAQNH
67	139	SWKPVLLSESLGADI	52	ASVHCGRYPAMAYRG	5	AWRSSDHYGAVQANK
68	138	SAWRITDIAKGDMGS	51	SLVSSHLLGPLGRGA	5	AVWTSRSTNVTVGQN
69	138	LLIGDYLRVNWFPRL	51	NTSCLGQNSPRAGLK	5	APARNFQPQLPLCNK
70	137	IKFPLYWVLGRPIYW	50	RGGSDAVRRPPCHIS	5	ALRFDPRRPWCKTHE
71	135	QWIAPIRKLIDRISV	50	ICKLDSSALMAGSPW	5	ALITKSSQPYVTSTL
72	135	PVCACHRLTNVVKHL	48	NRSVPSREPTTKHCS	5	ADQPIRRSIRYWFTS
73	135	MCALAIYESQHISSV	46	VVGKVSPNSMSGNHR	5	ACKRNTCSAEKLEGY
74	135	HTPFYTFYRPALGKA	46	SPLCCRLENHSNRGN	4	YYYQSHTWGHNSHIH
75	135	CSDLFTHLTIDRFTI	45	PSSTPGGITQRANTR	4	YYPQKKCPLLRLLSE
76	134	LCRALFSPTKTFFLI	43	GISRVSCVSRGPDIN	4	YYKQCSKPAGDPGCR
77	133	QCCLCGIRYWIRRRF	42	PSRNANSESRNCGSQ	4	YYGRPTSQPDCETRV
78	133	PFRSLPKSTVHSKCN	42	PLFSLSHLSHGNTHN	4	YVSSVLRLPAQKWTT
79	132	YYIVRKIVIIRNRFR	39	SGFVHHCLSAMSHSS	4	YVSEYWPSPTLPYTP
80	131	LPSHNVPNVGLWVLP	39	CSHCSFLTCKPIVKL	4	YVLVPSWLKDAGPRM
81	130	PYDFARRRTRGFRPN	38	SSNPVLPVRGDTKYK	4	YVLSPARTGDVMPAN
82	129	VIYPSSSMWFLSRAE	38	FAVREYGHHPAIVPY	4	YVHSTYRVSRYGAMK
83	129	SIARVFPLILVILHP	37	YTYVSPGVRGSTNQK	4	YVAARHSRGTATFTI
84	129	LISIFFWGDTPLSPC	37	LRDHNNVSYGRAALF	4	YTSIAPPGSDVTKER
85	129	CQCGAHALIIHCPFL	37	AKWRLSANHQAVSSL	4	YTRHTRKEVSGCSNK
86	129	CHSNIYCVAWHCIFQ	36	GRDTQFITTRPHDWC	4	YTLVALRESWRSCDE
87	128	TICMSFKNGLPGRRS	35	SFVATFSSIEKQPRC	4	YSSFLLPSSIYTANI
88	127	PIRRLSSSLNESRFM	35	LSRGTTCSLIHVNNV	4	YSNAVTLSESRLQAD
89	126	VSGLTAHDGGTGFHI	34	TRHGILTSYLFTGSS	4	YSMRQVQITPANVCA
90	126	TNSPVSIKARMIKEF	34	NREMPSSHSCDRQLN	4	YSGRAQGPTPGSYSG
91	126	GYINVLQCSKQSTCD	34	ALCDAAWNLHRLSKC	4	YRYPVYVADLPTGRK
92	125	IRFSTLFNQLRKSGC	34	AFRSKNYHWTFTGSS	4	YRTPYCSRHQLGLFN
93	124	YRTGVHTWNLLVLVR	33	YFHTPRPGSNSPWYP	4	YRTNFPGLSPEDNQL
94	124	VVWAHRRSRRRSLEW	33	VAFPSHWTKSPPYIQ	4	YRIPYLTSRLNKNSR
95	124	ACVGPSYATSTWHQN	33	SFKHAPNTGILMRST	4	YRHLDPGESIQAIFT
96	123	MSLRFHRNRPPLIIF	33	RTSPHHDLSYAHAGD	4	YPYELGNTKPILKLS
97	123	DSVIIRAMIATRSRL	32	TDCKYTYFPDVTSHG	4	YPKGGSHAEFSNTRQ
98	122	CALLVKHSITYRIGP	32	SRSQSVILHPLEPIT	4	YPHVSTLTRSFVHQG
99	121	CGIWPHTHSLTCYFT	32	PNPHVPGRDQYIVKH	4	YPFILQNLTSGRYQP
100	120	ITQFRYRLSKYALIG	32	MQHLSGSYSALLLNP	4	YNPDLTQLSEKSLHN

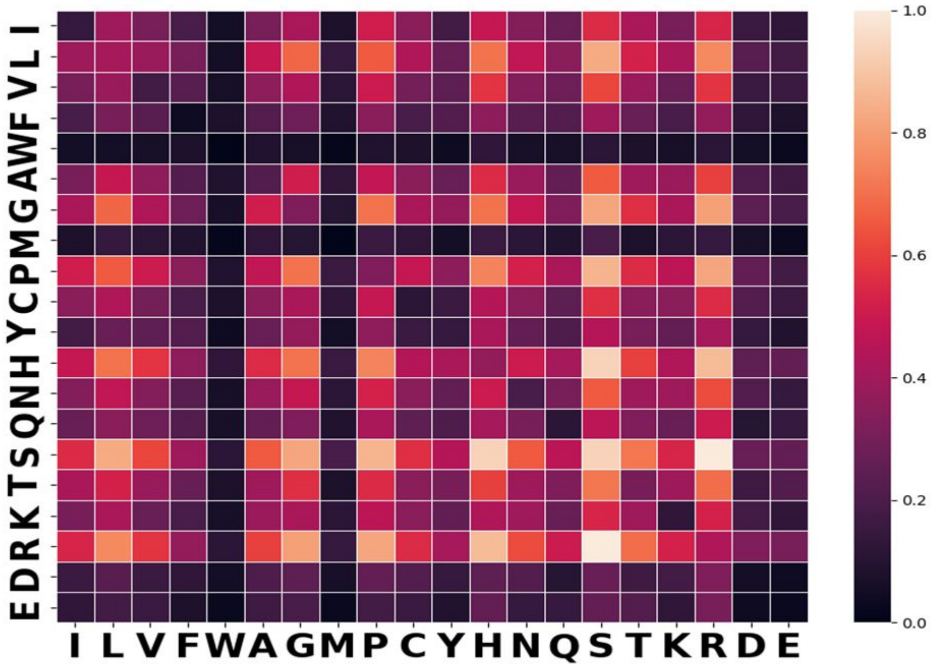

Figure S5. Heat Map of co-occurrence of amino acids in round 4 library sorting against gold.

Table S6. Identification of environmental microbes separated from loam and feedlot soil samples. The identification process was conducted by using nanopore sequencing.

Loam soil		Feedlot soil	
Number of microbe	Classification	Number of microbe	Classification
14	Stenotrophomonas sp. G4	2	Terribacillus goriensis
15	Stenotrophomonas sp. G4	9	Bacillus paralicheniformis
18	Stenotrophomonas sp. G4	16	Bacillus paralicheniformis
19	Stenotrophomonas sp. G4	18	Terribacillus goriensis
22	Enterobacter ludwigii (also possibly Kosakonia cowanii, or Enterbacter cloacae comples sp. FDA-CDC-AR_0132)	19	Bacillus safensis
26	Stenotrophomonas sp. G4	27	Bacillus pumilus
27	Stenotrophomonas sp. G4	28	Bacillus subtilis
32	Stenotrophomonas sp. G4	29	Bacillus sp. WP8
33	Stenotrophomonas sp. G4	30	Bacillus paralicheniformis
34	Stenotrophomonas maltophillia	39	Terribacillus goriensis
42	Stenotrophomonas sp. G4	40	Bacillus marisflavi
46	Stenotrophomonas sp. G4	41	Bacillus sp. WP8
